# Combination therapy targeting both innate and adaptive immunity improves survival in a pre-clinical model of ovarian cancer

**DOI:** 10.1186/s40425-019-0654-5

**Published:** 2019-07-30

**Authors:** Christina A. Hartl, Adrian Bertschi, Regina Bou Puerto, Carolin Andresen, Emily M. Cheney, Elizabeth A. Mittendorf, Jennifer L. Guerriero, Michael S. Goldberg

**Affiliations:** 1 Department of Cancer Immunology and Virology, Dana-Farber Cancer Institute, Boston, MA 02215 USA; 2 Breast Tumor Immunology Laboratory, Susan F. Smith Center for Women’s Cancers, Dana-Farber Cancer Institute, Boston, MA 02215 USA; 3 Division of Breast Surgery, Department of Surgery, Brigham and Women’s Hospital, Boston, MA 02215 USA; 4 Breast Oncology Program, Dana-Farber Cancer Institute, Boston, MA 02215 USA

**Keywords:** Ovarian cancer, Cancer immunotherapy, Combination therapy, CD4^+^ T cells, Innate immunity

## Abstract

**Background:**

Despite major advancements in immunotherapy among a number of solid tumors, response rates among ovarian cancer patients remain modest. Standard treatment for ovarian cancer is still surgery followed by taxane- and platinum-based chemotherapy. Thus, there is an urgent need to develop novel treatment options for clinical translation.

**Methods:**

Our approach was to analyze the effects of standard chemotherapy in the tumor microenvironment of mice harboring orthotopic, syngeneic ID8-Vegf-Defb29 ovarian tumors in order to mechanistically determine a complementary immunotherapy combination. Specifically, we interrogated the molecular and cellular consequences of chemotherapy by analyzing gene expression and flow cytometry data.

**Results:**

These data show that there is an immunosuppressive shift in the myeloid compartment, with increased expression of IL-10 and ARG1, but no activation of CD3^+^ T cells shortly after chemotherapy treatment. We therefore selected immunotherapies that target both the innate and adaptive arms of the immune system. Survival studies revealed that standard chemotherapy was complemented most effectively by a combination of anti-IL-10, 2′3’-cGAMP, and anti-PD-L1. Immunotherapy dramatically decreased the immunosuppressive myeloid population while chemotherapy effectively activated dendritic cells. Together, combination treatment increased the number of activated T and dendritic cells as well as expression of cytotoxic factors. It was also determined that the immunotherapy had to be administered concurrently with the chemotherapy to reverse the acute immunosuppression caused by chemotherapy. Mechanistic studies revealed that antitumor immunity in this context was driven by CD4^+^ T cells, which acquired a highly activated phenotype. Our data suggest that these CD4^+^ T cells can kill cancer cells directly via granzyme B-mediated cytotoxicity. Finally, we showed that this combination therapy is also effective at delaying tumor growth substantially in an aggressive model of lung cancer, which is also treated clinically with taxane- and platinum-based chemotherapy.

**Conclusions:**

This work highlights the importance of CD4^+^ T cells in tumor immunology. Furthermore, the data support the initiation of clinical trials in ovarian cancer that target both innate and adaptive immunity, with a focus on optimizing dosing schedules.

**Electronic supplementary material:**

The online version of this article (10.1186/s40425-019-0654-5) contains supplementary material, which is available to authorized users.

## Background

Epithelial ovarian carcinoma is the most lethal gynecological cancer, with around 22,240 new cases of ovarian cancer in 2018 and 14,070 deaths in the United States alone [1]. Despite major efforts invested in studying new cytotoxic and targeted agents, survival rates for ovarian cancer have increased only marginally in the last 40 years [2]. Standard treatment remains surgery and a combination of paclitaxel and carboplatin chemotherapy [3]. However, the success of cytotoxic chemotherapy is generally short-lived in patients. Almost invariably, the emergence of residual drug-resistant cells leads to recurrence after completion of therapy, as seen in about 75% of ovarian cancer patients [4].

Evidence presented over the last decade has shown that ovarian cancer is an immunogenic tumor that can be recognized by the host immune system [5]. Indeed, the first proof that the presence of intratumoral T cells correlates with improved clinical outcome was demonstrated in advanced ovarian cancer [6]. Also, antigen-specific antibodies and tumor-reactive T cells have been isolated from ovarian cancer patients [7]. The antitumor response mediated by the immune system is able to adapt to an evolving heterozygous cancer cell population and generate antitumor memory, which enables surveillance and elimination of minimal residual disease present even after completion of treatment.

Unfortunately, responses to immune checkpoint blockade to date remain modest in this patient population, with only ~ 15% overall response [8], as immune evasion by ovarian tumors often renders antitumor responses incomplete. Evidence is emerging that complementary therapy of chemotherapy and immunotherapy may yield a synergistic antitumor response and improve the magnitude and frequency of responses [9, 10]. Chemotherapy can generate antigenic debris in the context of danger signals, thereby producing an in situ vaccine [11]. Still, robust antitumor immunity is generally not achieved potentially because ovarian tumors have large numbers of regulatory T cells [12].

There is an urgent need to develop novel strategies for improving the outcomes of ovarian cancer patients. Current clinical trials in ovarian cancer have mostly focused on using immunomodulatory drugs that have been effective in other cancer types [13]. However, ovarian carcinomas have a unique tumor microenvironment [14] and treatments that benefit melanoma or bladder cancer patients may not be optimally suited for ovarian cancer patients. Therefore, our goal was to identify a mechanistically informed immunotherapy that synergizes with standard chemotherapy by discerning the impact of the chemotherapy on the immune compartment of the tumor microenvironment in an aggressive murine model of ovarian cancer.

In the orthotopic, syngeneic ID8-Vegf-Defb29 model of ovarian cancer, we found that chemotherapy induces acute immunosuppression mediated by cells of the innate immune system. We hypothesized that a single immunotherapeutic agent would not be sufficient to reverse the magnitude of this immunosuppression and therefore focused on immunotherapy combinations that could not only reduce suppression but also increase immune activation. Our results show that augmenting chemotherapy with anti-IL-10, 2′3’-cGAMP, and anti-PD-L1 can significantly increase survival compared to chemotherapy alone. This benefit is mediated by activated dendritic cells and T cells and is greatly influenced by the dosing schedule. Furthermore, our data show that CD4^+^ T cells are the main drivers of antitumor immunity. Importantly, our combination was effective not only against ovarian cancer but also in an aggressive model of lung carcinoma. Our hope for this work is to improve treatment options for ovarian cancer patients, with a view towards curative outcomes. More broadly, the approach hopefully underscores the utility of leveraging mechanistic insights into how standard therapy impacts the immune compartment to identify complementary combination immunotherapy.

## Methods

### Cell culture

ID8 murine ovarian cancer cells that overexpress VEGF-A and DEFB29 (kindly provided by Dr. Jose Conejo-Garcia, Moffitt Cancer Center and referred to as “ID8-Vegf-Defb29” within this manuscript) were grown in RPMI 1640 medium with 10% FBS, 1% penicillin-streptomycin, 1% L-glutamine, 0.5% sodium pyruvate, and 0.24 μM 2-mercaptoethanol. The murine Lewis Lung Carcinoma (LLC) lung cancer cell line (kindly provided by Dr. Harvey Cantor, Dana-Farber Cancer Institute, DFCI) were cultured in complete DMEM with 10% FBS, 1% penicillin-streptomycin, and 1% sodium pyruvate. Cells were sent out to Charles River Laboratories’ animal diagnostic services for mycoplasma testing using the mouse CLEAR Essential panel and found to be negative. All media supplements were obtained from Life Technologies.

### In vivo therapeutic experiments

Animal experiments were carried out in accordance with protocols approved by the DFCI Institutional Animal Care and Use Committee (IACUC). Six-week-old C57BL/6 female mice were purchased from Jackson Laboratory (Stock #000664). Mice were housed in the DFCI animal facility. Three million ID8-Vegf-Defb29 cancer cells (in 200 μl DPBS) were inoculated intraperitoneally (i.p.) into the mice. For the initial Nanostring and flow cytometry experiments, the mice were randomly assigned to treatment groups; 8 days after inoculation, mice were injected i.p. with vehicle control (0.5% DMSO + 15% polyethylene glycol + 0.5% Tween80 + ddH_2_O) or a combination of paclitaxel (15 mg/kg; Selleckchem) and carboplatin (20 mg/kg; Selleckchem) (referred to as “chemotherapy” within this manuscript). For the subsequent survival studies that included immunotherapy, mice were injected with vehicle control or chemotherapy 8 days after inoculation followed by i.p. administration of either isotype control antibody or various combinations of anti-IL-10 (0.25 mg/dose; clone JES5-2A5; BioXCell), 2′3’-cGAMP (0.01 mg/dose; Invivogen), anti-PD-L1 (0.2 mg/dose; clone 10F.9G2; BioXCell), gemcitabine (1.2 mg/dose; Selleckchem), anti-4-1BB (0.1 mg/dose; clone 3H3; BioXCell), GR-MD-02 (1.2 mg/dose; Galectin Therapeutics). A detailed description of the treatment schedule for each experiment is provided in the figure legends. Tumor growth was measured using body weight and mice were sacrificed when body weight reached 150% or mice became moribund. For experiments involving the LLC lung cancer cell line, mice were inoculated subcutaneously with 1 × 10^6^ cells (in 100 μl DPBS) to generate a local tumor mass. Mice were randomly assigned to treatment groups and received (i) vehicle control, (ii) paclitaxel and carboplatin, (iii) anti-IL-10, 2′3’-cGAMP, and anti-PD-L1, or (iv) paclitaxel, carboplatin, anti-IL-10, 2′3’-cGAMP, and anti-PD-L1 when tumors reached ~ 100 mm^3^ (about 16 days after tumor inoculation). Tumor volume was measured using electronic calipers, and the volume was calculated using the formula (L x W^2^)/2. Studies were performed in duplicate and included at least 10 mice per group.

### Cell isolation, cell sorting, and Nanostring

Cells were harvested from the peritoneal cavities of mice by peritoneal wash. Briefly, 5 ml ice-cold DPBS + 3% FBS was injected into the peritoneal cavity, the peritoneum was gently massaged, and the fluid containing peritoneal cells was collected through a 21G needle and placed on ice. Red blood cells were removed by ACK buffer (Life Technologies cat. A1049201) and cells were stained with Zombie Aqua Fixable Viability Kit (BioLegend cat. 423,101) and anti-mouse CD16/32 antibody (BioLegend cat. 101,302, clone 93) was added to block interactions with Fc. Subsequently cells were stained for anti-mouse CD45 PerCP/Cy5.5 (BioLegend cat. 103,131, clone 30-F11), anti-mouse CD3 APC (BioLegend cat. 100,236, clone 17A2), anti-mouse CD11b FITC (BioLegend cat. 101,205, clone M1/70), anti-mouse B220 PE (BioLegend cat. 103,207, clone RA3-6B2), and anti-mouse NKp46 PE (BioLegend cat. 137,603, clone 29A1.4). Cells were then sorted on a BD FACSAria as ZombieAqua^−^/CD45^+^/CD3^+^/CD11b^−/−^/B220^−^/NKp46^−^ or ZombieAqua^−^/CD45^+^/CD3^−^/CD11b^+^/B220^−^/NKp46^−^ cells into RPMI 1640 medium containing 2% FBS at 4 °C. Cells were pelleted, and RNA was isolated using the PureLink RNA Mini Kit (Ambion cat. 12183018A) according to manufacturer’s instructions. RNA quality was verified with the Nanodrop Spectrophotometer, and 100 ng of RNA per sample was loaded and run on the MmV1_CancerImm_CSO-MIP1–12 Nanostring instrument for analysis of the NanoString PanCancer Immune Profiling Panel (NanoString Technologies). Sample were analyzed using the Advanced Analysis Module of the nSolver™ software (NanoString Technologies). In short, samples were normalized against positive controls and selected housekeeping genes using the geometric mean. Ideal normalization genes were determined automatically by selecting those that minimize the pairwise variation statistic. Differential expression to identify specific targets was performed, and *p*-values were adjusted using the Benjamini-Hochberg procedure.

### Flow cytometry

Cells were harvested from the peritoneal cavity by peritoneal wash as described above. Red blood cells were removed by ACK buffer (Life Life Technologies cat. A1049201) and cells were stained with Zombie Aqua Fixable Viability Kit (BioLegend cat. 423,101). Anti-mouse CD16/32 antibody (BioLegend cat. 101,302, clone 93) was added to block interactions with Fc. Cell Activation Cocktail with Brefeldin A (BioLegend cat. 423,304) and GolgiStop™ protein transport inhibitor (BD Biosciences cat. 554,724) were used for inspection of intracellular cytokines and cytolytic molecules. Flow cytometry was performed on a Sony SP6800 Spectral Analyzer (Sony Biotechnology), and all antibodies were purchased from BioLegend, R&D Systmes, or Cell Signaling Technology (listed in Additional file 14: Table S1).

### Depletion of CD4^+^ T cells, CD8^+^ T cells, or CD11b^+^ cells

In order to evaluate which immune cells are required to confer the observed anti-tumor effect, specific cell subsets (CD4^+^ T cells, CD8^+^ T cells, or CD11b^+^ cells) were depleted by administering depleting antibodies i.p., beginning 1 day prior to chemotherapy. The antibodies used for depletion were anti-mouse CD4 (BioXCell cat. BE0003–1, clone GK1.5), anti-mouse CD8a (BioXCell cat. BE0061, clone 2.43), and anti-mouse CD11b (BioLegend cat. 101,231, clone M1/70). Two-hundred μg of anti-CD4 or anti-CD8a was administered every 3 days, or 100 μg of anti-CD11b was administered every 2 days. Depletion of CD4^+^ T cells, CD8^+^ T cells, and CD11b^+^ cells was confirmed by flow cytometry of leukocytes isolated from the blood of mice to which antibodies or isotype antibody (BioXCell cat. BE0090, clone LTF-2) had been administered.

### Statistical methods

Statistical methods were not used to predetermine necessary sample size. The sample sizes were selected based on the results of pilot experiments so that relevant statistical tests could reveal significant differences between experimental groups. Statistical analysis was performed using GraphPad Prism software version 7.01. Data are presented as mean ± SEM, as indicated in the Figure legends. The Student’s t-test or one-way ANOVA with Tukey’s post-hoc test was used to determine statistical significance between two groups and several groups, respectively. For survival analysis, the Log-rank (Mantel-Cox) test was employed. * *p* ≤ 0.05, ** *p* ≤ 0.01, *** *p* ≤ 0.001, **** *p* ≤ 0.0001.

## Results

### Chemotherapy induces acute immunosuppression specifically among innate immune cells

In this study, we examined the effects of standard chemotherapy on the peritoneal immune compartment of mice harboring ovarian cancer. These insights were sought to enable identification of a mechanistically informed immunotherapy that should combine synergistically with chemotherapy and thereby increase overall survival. We selected the orthotopic, syngeneic ID8-Vegf-Defb29 ovarian cancer model in C57BL/6 J mice because it is an aggressive variant of the parental ID8 cell line that robustly recapitulates many features of advanced human ovarian cancer [15]. Consistent with clinical presentation, ID8-Vegf-Defb29 tumors grow throughout the peritoneal cavity in small nodules and lead to severe ascites formation at a late stage. Treatment with chemotherapy alone is not curative in this model, which also exhibits low sensitivity to combination therapy with checkpoint blockade alone (Additional file 1: Figure S1a, b).

**Fig. 1:**
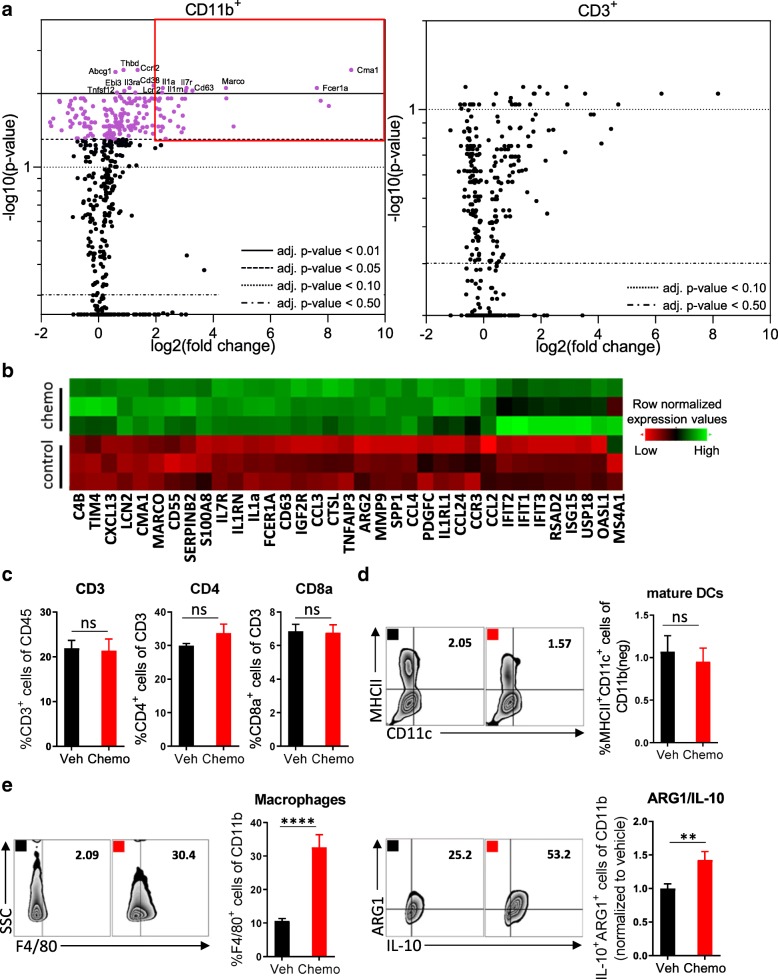
Treatment with paclitaxel and carboplatin induces acute immunosuppression that is mediated by innate immune cells. Mice were inoculated orthotopically with ID8-Vegf-Defb29 ovarian cancer cells. Eight days later, the mice were injected with vehicle (Veh) or chemotherapy (Chemo). Two days later, peritoneal cells were harvested for analysis. **a** Volcano plots of gene expression data sets derived from FACS-sorted leukocytes (CD11b^+^ and CD3^+^). All probe sets are shown. The top differentially expressed genes in the myeloid population are named, and highlight coloring was applied to significantly differentially expressed (adj. *p*-value < 0.05) probe sets. The experiment was performed once with *n* = 3 biological replicates. **b** A heatmap of the top 35 upregulated genes after chemotherapy treatment in FACS-sorted CD11b^+^ cells. **c** Peritoneal cell suspensions were assessed by flow cytometry. Bar graphs show quantification of flow cytometry gating of CD3^+^ T cells, CD4^+^ T cells, and CD8^+^ T cells. **d** Flow cytometry gating of subsets of MHCII^+^ mature dendritic cells are shown as scatter plots and quantified at right. **e** Flow cytometry gating of subsets of F4/80^+^ macrophages are shown as scatter plots and quantified at right. Increased numbers of immunosuppressive ARG1^+^ IL-10^+^ myeloid cells are observed following chemotherapy. The experiment was performed twice with *n* = 4 biological replicates. Statistics were calculated using a two-sided unpaired t-test. Data are presented as mean ± SEM * *p* ≤ 0.05, ** *p* ≤ 0.01, **** *p* ≤ 0.0001

Peritoneal leukocytes were harvested from tumor-bearing mice 2 days after treatment with a single dose of paclitaxel and carboplatin; a standard regimen used to treat ovarian cancer patients. This time point was chosen to inspect the short-term consequences of chemotherapy on the immune system, as we sought to initiate immunotherapy concomitantly in order to leverage the benefits of chemotherapy and mitigate against its drawbacks. Nanostring-mediated analysis of FACS-sorted CD11b^+^ myeloid cells or CD3^+^ lymphocytes revealed a selective induction of differential gene expression in myeloid cells (Fig. 1a; Additional file 2: Figure S2). Among CD11b^+^ cells, mRNA expression was increased for 200 genes, 35 of which were upregulated more than 2-fold (Fig. 1b). In contrast, no significantly differential gene expression was detected among CD3^+^ T cells using an adjusted *p*-value of 0.05 or lower. Flow cytometric analysis of peritoneal leukocytes confirmed that chemotherapy predominantly affected the myeloid compartment, as evidenced by a lack of change in the proportion of CD3^+^, CD4^+^, and CD8^+^ T cells (Fig. 1c; Additional file 3: Figure S3) and mature dendritic cells (MHCII^+^) (Fig. 1d). Consistently, the number of granulocytic MDSCs (Ly6G^+^/Ly6C^+^) (Additional file 4: Figure S4a), was increased, and the proportion of macrophages (F4/80^+^) and CD11b^+^ cells that expressed the immunosuppressive factors ARG1 and IL-10 [16–18] was similarly elevated (Fig. 1e). In contrast, the number of monocytic MDSCs (Ly6G^−^/Ly6C^+^) and their expression levels of ARG1 and IL-10 did not change (Additional file 4: Figure S4b). Together, these data indicate that chemotherapy induces acute immunosuppression in this model.

### STING agonism combined with neutralization of IL-10 and PD-L1 after chemotherapy increases survival

To identify an immunotherapy that best synergizes with paclitaxel and carboplatin, we compared the relative efficacy of several immunotherapy combinations. To stimulate the adaptive arm of the immune system, we selected anti-PD-L1, which enhances cytotoxic function [19], and an agonist of 4-1BB, a co-stimulatory receptor and important regulator of immune responses [20]. Neutralization of the PD-1 pathway is likely to be the backbone of immunotherapy for treatment of ovarian cancer [21]; however since anti-PD-(L)1 monotherapy of ovarian cancer is inadequate in the clinic [8] and completely ineffective in combination with chemotherapy in preliminary experiments in the ID8-Vegf-Defb29 model (Additional file 1: Figure S1b), we decided to simultaneously target the innate immune system.

**Fig. 2:**
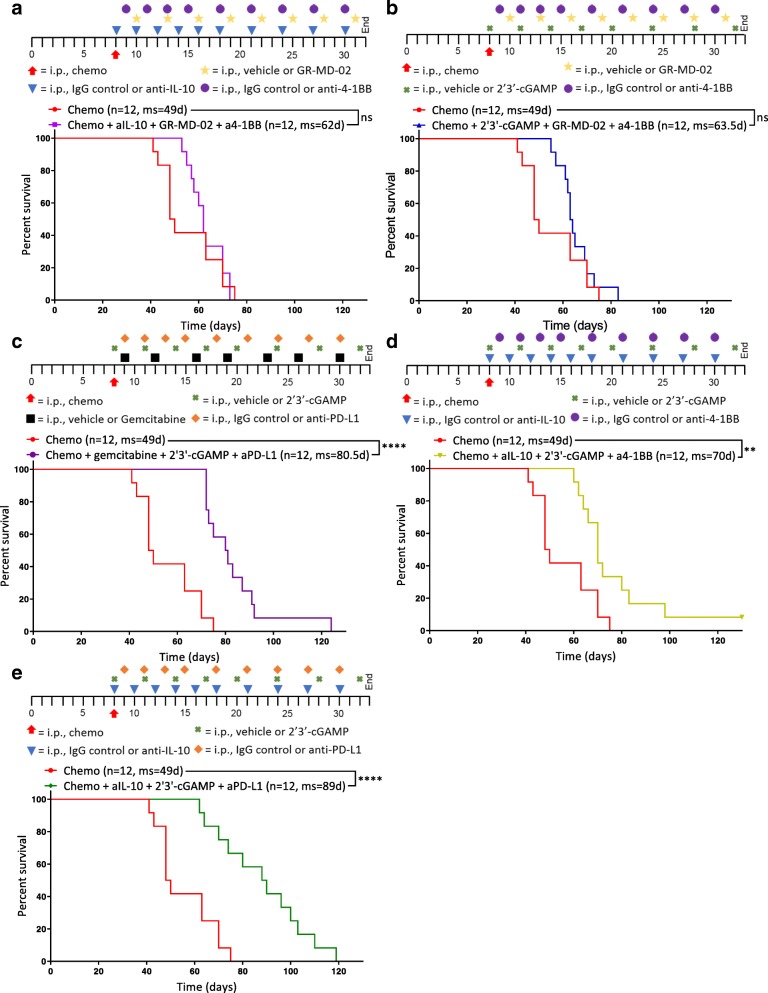
STING agonism combined with neutralization of IL-10 and PD-L1 after chemotherapy increases survival. Different combinations of chemotherapy and immunotherapy were tested in vivo for synergy. Kaplan-Meier curves are shown for mice treated with chemotherapy and (**a**) anti-IL-10, GR-MD-02, and anti-4-1BB, **b** 2′3’-cGAMP, GR-MD-02, and anti-4-1BB (**c**) gemcitabine, 2′3’-cGAMP, and anti-PD-L1, **d** anti-IL-10, 2′3’-cGAMP, and anti-4-1BB, or (**e**) anti-IL-10, 2′3’-cGAMP, and anti-PD-L1. **a-e** All combination treatments were compared to chemotherapy and isotype control for immunotherapy (Chemo) 8 days after inoculation of ID8-Vegf-Defb29 cells. The number of mice per group (n) and median survival (ms) are listed. The experiment was performed with biological replicates twice. Statistics were calculated relative to the group treated with chemotherapy only using the Log-rank (Mantel-Cox) test. ** *p* ≤ 0.01, **** *p* ≤ 0.0001

Thus, as complement to the adaptive immunotherapy, we tested inhibitors of interleukin-10 (anti-IL-10) and Galectin-3 (GR-MD-02), two negative regulators of immune function [18, 22] whose expression and MFI were respectively upregulated on myeloid cells, as determined by flow cytometry (Fig. 1e, Additional file 5: Figure S5). Gemcitabine is a chemotherapy known to preferentially deplete immunosuppressive MDSCs [23], and 2′3’-cGAMP is an agonist of Stimulator of interferon genes (STING) that potently induces production of type I interferons [24]. GR-MD-02 and agonist anti-4-1BB were combined with either anti-IL-10 or 2′3’-cGAMP. Anti-PD-L1 and 2′3’-cGAMP were combined with gemcitabine or anti-IL-10. 2′3’-cGAMP and anti-IL-10 were combined with an activator of the adaptive immune system: anti-PD-L1 or agonist anti-4-1BB. Immunotherapies were administered promptly after chemotherapy into tumor-bearing mice and dosed as described (see Methods; Fig. 2, Additional file 15: Table S2). Paclitaxel and carboplatin in the absence of immunotherapy (Chemo) were administered as a control.

Tumor burden was monitored using ascites as a surrogate for disease progression, and it was confirmed that combination of immunotherapy and chemotherapy can significantly extend survival in some groups relative to chemotherapy only control (Fig. 2a-e). Notably, not all combinations increased survival equally.

GR-MD-02, which inhibits M2 macrophage polarization and angiogenesis, had little impact relative to anti-IL-10 and 2′3’-cGAMP (Fig. 2a, b, d). Gemcitabine provided some benefit but was inferior to anti-IL-10 (Fig. 2c, e). As a complement to anti-IL-10 and 2′3’-cGAMP, anti-PD-L1 conferred greater survival benefit than agonist anti-4-1BB (Fig. 2d, e). These data suggest that both the neutralization of immunosuppressive cytokines (anti-IL-10) anti-IL-10 and 2′3’-cGAMP and the induction of an inflammatory innate immune response (2′3’-cGAMP) are essential for establishing meaningful antitumor immunity following chemotherapy. Furthermore, the increased survival conferred by anti-PD-L1 therapy (Fig. 2e) suggests an essential role of T cells in mediating anti-tumor effects, though this effect is likely enabled by the continued dosing of the antibody beyond the neuralization of acute immunosuppression. These results suggest that immunotherapy targeting both innate and adaptive immune function generated the greatest survival benefit. We therefore selected anti-IL-10, 2′3’-cGAMP, and anti-PD-L1 as immunotherapy combination for all subsequent experiments.

### Combination therapy reverses the myeloid cell-mediated immunosuppression and promotes infiltration of activated DCs and T cells

**Fig. 3:**
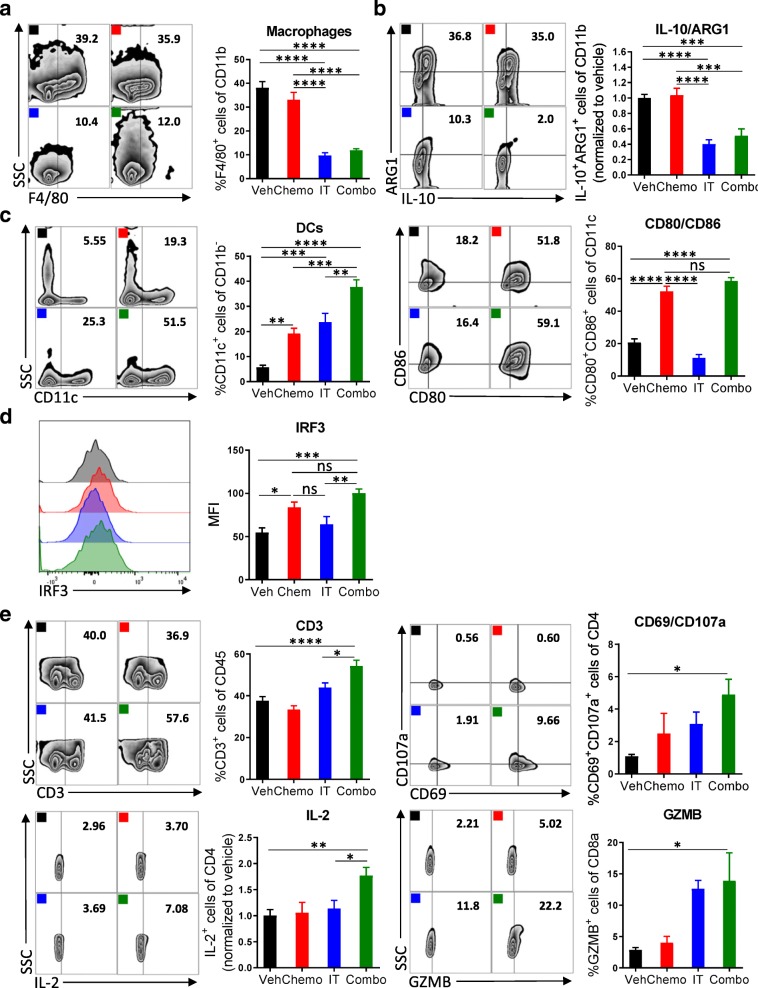
Combination therapy reverses the myeloid cell-mediated immunosuppression and promotes infiltration of activated DCs and T cells. **a** Peritoneal cell suspensions from tumor-bearing mice treated with vehicle (Veh); chemotherapy (Chemo); anti-IL-10, 2′3’-cGAMP, and anti-PD-L1 immunotherapy (IT); or both Chemo and IT (Combo) were assessed by flow cytometry 4 days after initiation of treatment. **a, b** Decreased numbers of myeloid cells with immunosuppressive phenotypes are observed upon Combo treatment. **a** Decreased numbers of F4/80^+^ macrophages are observed upon treatment with immunotherapy (IT and Combo) (**b**) Flow cytometry gating of subsets of ARG1^+^IL-10^+^ myeloid cells are shown as scatter plots and quantified at right. **c, d** Increased numbers of mature dendritic cells are observed upon Combo treatment. **c** Flow cytometry gating of subsets of CD11c^+^ dendritic cells are shown as scatter plots and quantified at right. Numbers of CD11c^+^ cells expressing co-stimulatory molecules are quantified. **d** STING activation is pharmacodynamically confirmed by increased median fluorescence intensity of IRF3. **e** The adaptive immune system is also impacted by Combo therapy. Flow cytometry gating of subsets of CD3^+^ T cells are shown as scatter plots and quantified at right. Increased numbers of CD4^+^ T cells expressing the activation marker CD69, the cytolytic molecule CD107a, and the pro-inflammatory cytokine IL-2 are observed. Increased numbers of CD8^+^ T cells expressing the cytolytic molecule GZMB are shown. The experiment was performed twice with *n* = 4 biological replicates. Statistics were calculated using one-way ANOVA with Tukey’s multiple comparisons test. Data are presented as mean ± SEM * *p* ≤ 0.05, ** *p* ≤ 0.01, *** *p* ≤ 0.001, **** *p* ≤ 0.0001

To dissect the changes among immune cell subsets following administration of combination therapy on a cellular and molecular level, we assessed the immune cells recovered from the peritoneal cavity for expression of lineage and activation markers. Leukocytes were recovered from mice 4 days after initiation of treatment for flow cytometric analysis. We observed a significant decrease in macrophage numbers (CD11b^+^F4/80^+^) after treatment with immunotherapy (Fig. 3a). Similarly, the numbers of ARG1^+^ and IL-10^+^ myeloid cells, which are highly immunosuppressive, were decreased (Fig. 3b). After exposure to the combination of chemotherapy and immunotherapy, more dendritic cells were present in the tumor microenvironment, which were highly activated by chemotherapy as indicated by the elevated expression of costimulatory molecules CD86 and CD80 (Fig. 3c). Furthermore, an increased MFI of IRF3, a transcription factor in the STING pathway [25], suggested activation by chemotherapy as well as 2′3’-cGAMP [26] (Fig. 3d). Likely, this activation of dendritic cells translated into the robust T cell priming as evidenced by a strong adaptive antitumor response. The number of CD3^+^ T cells was increased after treatment with combination chemotherapy and immunotherapy, but not after either therapy alone or vehicle (Fig. 3e). While the numbers of CD4^+^ and CD8^+^ T cells did not change (Additional file 6: Figure S6), increased expression of the early activation marker CD69, the degranulation marker CD107a, the cytokine IL-2, and the cytolytic molecule granzyme B (GZMB) were detected (Fig. 3e, Additional file 7: Figure S7). The relatively short time between treatment and analysis might explain why significant changes in expression of IFNγ or PD-1 were not observed (Additional file 8: Figure S8). Together, these results indicate that combination of immunotherapy targeting both the innate and adaptive arms of the immune system can reverse the immunosuppressive phenotype of myeloid cells induced by chemotherapy and can commensurately lead to activation of T cells.

### Survival benefit of combination therapy is strongly influenced by dosing schedule

**Fig. 4:**
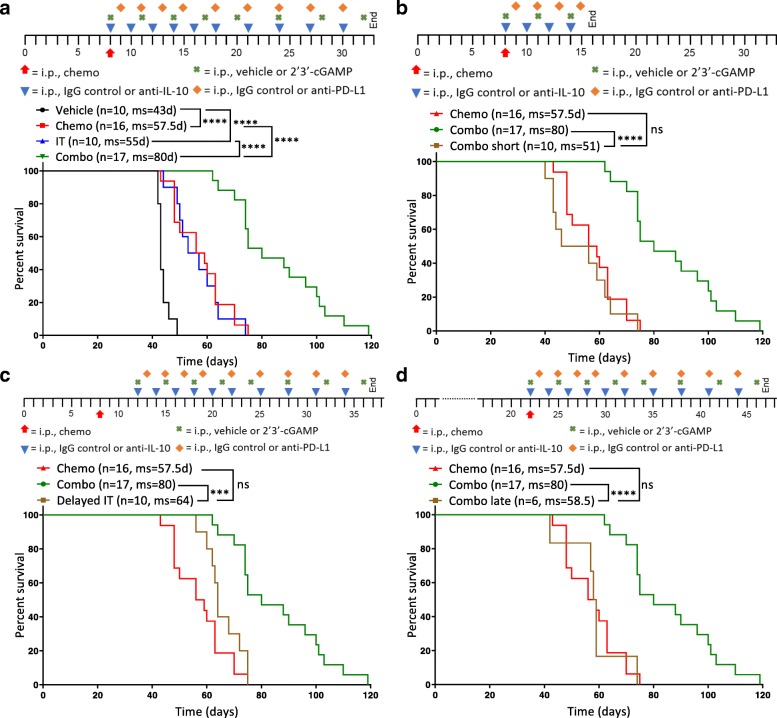
Survival benefit conferred by the combination therapy is superior to chemotherapy or immunotherapy alone and strongly influenced by dosing schedule**.** Different dosing schedules were tested to understand the temporal interaction between chemotherapy and immunotherapy in ID8-Vegf-Defb29-tumor bearing mice. Each is depicted above the Kaplan-Meier curves. **a** A Kaplan-Meier curve is shown comparing combination therapy (Combo) to chemotherapy (Chemo) or immunotherapy (IT) alone as well as vehicle only (Vehicle). **b** A Kaplan-Meier curve is shown comparing 3 weeks of treatment (Combo) to 1 week of immunotherapy treatment (Combo short) following chemotherapy. **c** A Kaplan-Meier curve is shown comparing immunotherapy initiated on the same day as chemotherapy (Combo) to immunotherapy initiated 4 days later (Delayed IT). **d** A Kaplan-Meier curve is shown comparing combination therapy initiated on day 8 (Combo) to combination therapy initiated on day 22 (Combo late). **b-d** Treatment groups are compared to chemotherapy and isotype control (Chemo). The number of mice per group (n) and median survival (ms) are listed. All experiments were performed with biological replicates at least twice. Statistics were calculated using the Log-rank (Mantel-Cox) test. *** *p* ≤ 0.001, **** *p* ≤ 0.0001

Next, we confirmed that chemotherapy and immunotherapy indeed work synergistically by comparing the combination of chemotherapy plus immunotherapy (Combo) to separate therapy with paclitaxel and carboplatin (Chemo) or anti-IL-10, 2′3’-cGAMP, and anti-PD-L1 immunotherapy (IT). Studies confirmed that while each therapy (chemotherapy/immunotherapy) alone significantly improves survival, the combination imparted a much larger benefit (Fig. 4a). Initial repolarization of the immune compartment can sometimes be sufficient to increase survival and enhance the antitumor effects of chemotherapy. To determine if prolonged immunotherapy is necessary for efficacy, we dosed mice with the combination for either the full 3 weeks (Combo) or just 1 week (Combo short). Dosing for only 1 week completely abrogates the survival benefit of the combination (Fig. 4b), suggesting that merely repolarizing the immune environment shortly after chemotherapy is not adequate and highlighting the importance of directly augmenting the adaptive immune system thereafter. It is thus possible that continued immunotherapy – beyond 3 weeks – could potentially further increase survival or even be curative.

Next, we investigated the importance of the early repolarization phase and the temporal interplay between chemotherapy and immunotherapy dosing. We dosed mice with chemotherapy on day 8 post-tumor inoculation in combination with immunotherapy beginning on day 8 (Combo) or day 12 (Delayed IT). We chose a delay of 4 days to minimize the possibility that any effects on survival would be caused by a dearth of therapy early in the course of disease progression, as might be expected if the therapy was delayed by 1 week or more. Still, a delay of just 4 days was sufficient to abolish the benefit of the combination therapy (Fig. 4c), supporting the notion that the immunosuppressive effects of chemotherapy are acute and that immediate intervention with immunotherapy is essential. This highlights the importance of a well-designed treatment schedule in the clinic to maximize patient outcome. In the clinic, ovarian cancer is often diagnosed at a late stage when patients have already developed extensive primary tumors and metastasis [27]. Therefore, we investigated whether our combination would have the same survival benefit when given to mice at a relatively late stage of cancer progression. Mice were treated with combination therapy beginning either on day 8 (Combo) or day 22 (Combo late). Results show that mice treated later do not benefit from the combination therapy (Fig. 4d). These data therefore suggest that this immunotherapy regimen works synergistically with chemotherapy in this model but that the dosing schedule is crucial to conferring benefit. Furthermore, the largest survival benefit is achieved when immunotherapy is given concomitant with chemotherapy at an early stage of disease for an extended period of time.

### CD4^+^ T cells are critical for the efficacy of this combination therapy

**Fig. 5:**
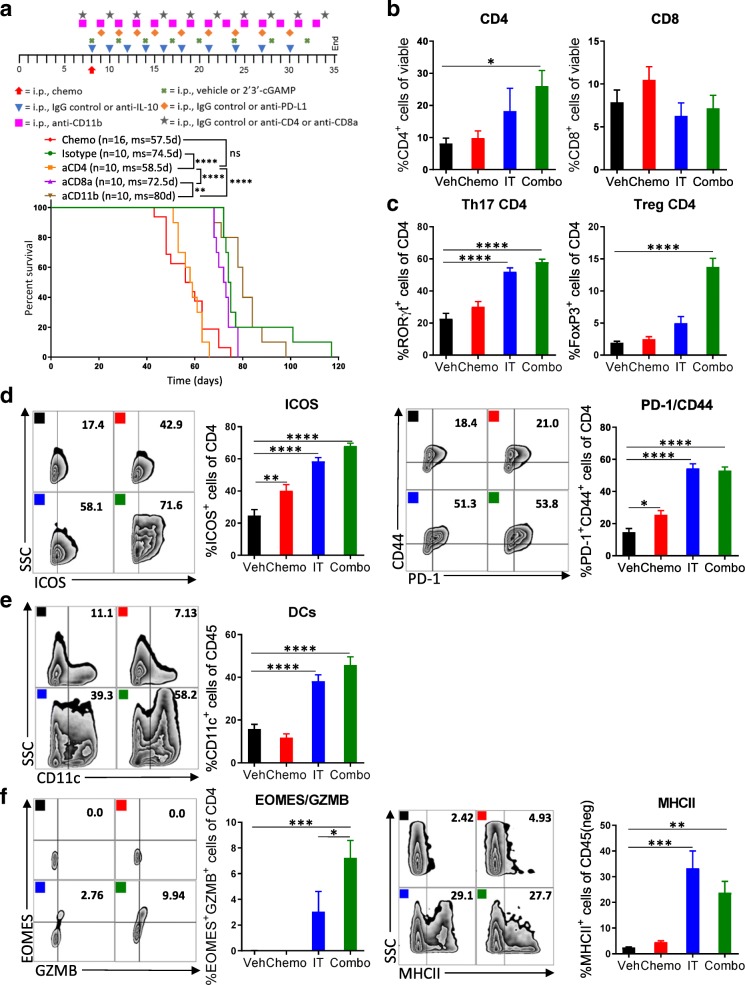
CD4^+^ T cells are critical for the efficacy of the combination therapy. **a** Specific immune cell subsets (CD4^+^ T cells, CD8^+^ T cells, or CD11b^+^ cells) were depleted to explore their relative contribution to the observed efficacy. Kaplan-Meier curves are shown for all groups described compared to isotype control. The number of mice per group (n) and median survival (ms) are listed. All experiments were performed twice with *n* = 5 biological replicates. Dosing schedule is shown at the top of the figure. Statistics were calculated using the Log-rank (Mantel-Cox) test. ** *p* ≤ 0.01, **** *p* ≤ 0.0001. **b-f** Peritoneal cell suspensions from tumor-bearing mice treated with vehicle (Veh); chemotherapy (Chemo); anti-IL-10, 2′3’-cGAMP, and anti-PD-L1 immunotherapy (IT); or both Chemo and IT (Combo) were assessed by flow cytometry 13 days after initiation of treatment. (**b**) Bar graphs show quantification of flow cytometry gating of CD4^+^ and CD8^+^ T cells. (**c**) Increased numbers of RORγt- and FoxP3-expressing CD4^+^ T cells are observed with Combo therapy. (**d**) CD4^+^ T cells expressing activation markers are observed. (**e**) Increased numbers of dendritic cells are observed upon Combo treatment even at this late timepoint. (**f**) Flow cytometry gating of subsets of GZMB expressing CD4^+^ T cells are shown as scatter plots and quantified at right. MHCII-expression on cancer cells is confirmed. The experiment was performed twice with *n* = 4 biological replicates. Statistics were calculated using one-way ANOVA with Tukey’s multiple comparisons test. Data are presented as mean ± SEM * *p* ≤ 0.05, ** *p* ≤ 0.01, *** *p* ≤ 0.001, **** *p* ≤ 0.0001

Having shown that immunotherapy activates both innate and adaptive immune cells, we subsequently sought to investigate the mechanistic pathway and effector cells underlying the enhanced antitumor immune response upon combination therapy. To this end, we treated mice with combination therapy and additionally depleted CD11b^+^ cells, CD8^+^ T cells, or CD4^+^ T cells (Additional file 9: Figure S9). Survival studies indicated that only CD4^+^ T cells are required for antitumor response (Fig. 5a). Mice whose CD4^+^ T cells had been depleted failed to benefit from combination therapy.

To dissect the cellular and molecular changes among CD4^+^ T cells after immunotherapy, we harvested leukocytes in the peritoneal cavity after 13 days of combination treatment and assessed their phenotype and function status with a focus on CD4^+^ T cells. In line with the survival studies, we saw a 3-fold increase in the proportion of CD4^+^ T cells with combination therapy, while the percentage of CD8^+^ T cells was unchanged (Fig. 5b). Looking into the phenotype of these CD4^+^ T cells, we found that immunotherapy alone or in combination caused a highly significant increase in Th17 cells, as indicated by expression of the transcription factor RORγt (Fig. 5c). Interestingly, the percentage of regulatory FoxP3^+^ CD4^+^ T cells among total CD4^+^ T cells was also significantly increased with combination therapy (Fig. 5c). In contrast, the involvement of T-bet-expressing Th1 cells in mediating antitumor immunity in this model is likely minor, as numbers were found to be low overall and not impacted by combination therapy (Additional file 10: Figure S10). The proportion of CD4^+^ T cells expressing ICOS, CD44, and PD-1 were markedly elevated by immunotherapy, indicating that these cells are antigen-experienced and highly active (Fig. 5d). When looking for cells that could potentially mediate this CD4^+^ T cell antitumor immunity, we observed a 2.5-fold increase in dendritic cells (Fig. 5e) and in mature dendritic cells (CD11c^+^MHCII^+^) (Additional file 11: Figure S11).

CD4^+^ T cells have several means by which to kill cancer cells. It has been previously shown that they can kill cancer cells directly through granzyme-dependent cytotoxic activity [28]. Indeed, combination therapy induces significant expression of GZMB and EOMES by CD4^+^ T cells, and immunotherapy alone or in combination with chemotherapy increased the proportion of epithelial cancer cells that expressed MHCII (Fig. 5f). These results indicate that CD4^+^ T cells are essential for extending survival in this model and that antitumor immunity is likely mediated by both Th17 helper cells as well as GZMB^+^EOMES^+^ cytotoxic CD4^+^ T cells.

### Efficacy of this combination therapy is similarly exhibited in a subcutaneous lung cancer model

**Fig. 6:**
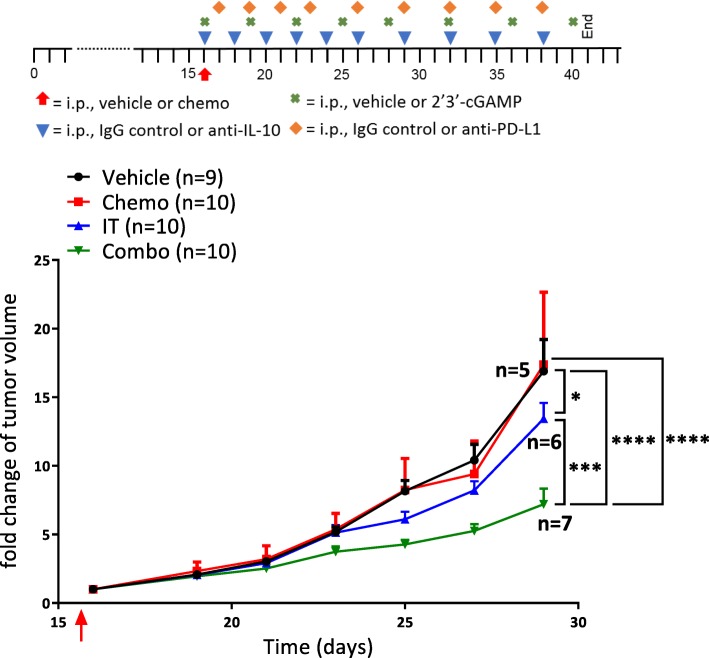
Efficacy of combination therapy is similarly observed in a subcutaneous lung cancer model. Combination therapy was tested in the murine LLC lung cancer model. Tumors were allowed to grow to an average of 100mm^3^ per group before initiation of treatment (red arrow). Average fold change of tumor volume of mice treated with combination therapy (Combo), chemotherapy (Chemo) alone, or immunotherapy (IT) alone as well as vehicle only (Vehicle). The number of mice per group (n) are listed. All experiments were performed with biological replicates twice. Statistics were calculated using two-way ANOVA and the Log-rank (Mantel-Cox) test. * *p* ≤ 0.05, *** *p* ≤ 0.001, **** *p* ≤ 0.0001

To test the efficacy of this new combination therapy in a second solid tumor model, the treatment was administered to mice harboring established Lewis Lung Carcinoma (LLC) tumors. Like ovarian cancer, lung carcinomas are routinely treated with paclitaxel and carboplatin in the clinic [29]; however, lung cancer exhibits a different tumor microenvironment, so it was not obvious that the combination would be similarly effective in this context. Tumors were allowed to grow to roughly 100 mm^3^ prior to commencement of therapy: paclitaxel and carboplatin (Chemo); anti-IL-10, 2′3’-cGAMP, and anti-PD-L1 immunotherapy (IT); chemotherapy plus immunotherapy (Combo); or control (Vehicle). Tumor volume measurements confirmed that chemotherapy alone had no influence on tumor growth, immunotherapy alone was able to delay tumor growth, and combination therapy had by far the largest benefit (Fig. 6). These results suggest that the combination treatment of chemotherapy and anti-IL-10, 2′3’-cGAMP, and anti-PD-L1 has the potential to slow tumor growth in aggressive forms of cancer.

## Discussion

In this study, we identified a complementary combination immunotherapy that, when administered together with standard chemotherapy, is able to significantly extend the survival of mice harboring aggressive models of ovarian cancer and lung cancer. The components of the combination were selected based on analysis of gene expression and flow cytometry data. It was further determined that the temporal interplay between chemotherapy and immunotherapy was as important as the components.

To achieve substantial survival benefit in the orthotopic ID8-Vegf-Defb29 ovarian cancer model, we found that chemotherapy had to be complemented by immunomodulators targeting both the innate and adaptive arms of the immune system. While it has been previously shown that paclitaxel can be immunostimulatory [30], carboplatin – a DNA adduct-forming compound – is generally administered in combination with this drug. Following injection of both cytotoxic compounds, we observed an immunosuppressive shift in the tumor microenvironment, as denoted by an increased myeloid cell population shortly after chemotherapy.

Immune checkpoint blockade is a powerful means by which to enhance the antitumor activity of T cells, and previous work in murine models of ovarian cancer has demonstrated the efficacy of PD-1 pathway blockade when combined with blockade of another immune checkpoint or with a vaccine [31, 32]. Unfortunately, these treatment regimens have not been observed to be similarly effective when translated to patients [13, 33, 34]. Factors that may account for the differential responses include the inability to recreate the orthotopic tumor microenvironment upon inoculating cancer cells subcutaneously as well as the use of a much less aggressive model that does not recapitulate the clinical setting quite as well. Our approach improves on past research by selecting a more realistic cancer model that is inoculated orthotopically.

Ovarian cancer often involves a highly immunosuppressive milieu that includes anti-inflammatory cytokines and a dearth of effector T cells [35]. Therefore, successful treatment of ovarian cancer in the clinic may require immunotherapy combinations that are able to stimulate antigen presenting cells, attenuate the immunosuppressive microenvironment, and enhance T cell stimulation and functionality. Consistent with what has been reported from early clinical studies [8], our work shows that PD-1 pathway blocking is largely ineffective as monotherapy for treatment of ovarian cancer. However, anti-PD-L1 therapy can be highly effective if it is combined with chemotherapy and other immunotherapies that address the innate arm of the immune system.

We hypothesize that neutralization of IL-10 in conjunction with production of type I interferons (IFNs) – induced by 2′3’-cGAMP-mediated activation of the STING pathway – reverses the tumor microenvironment from immunosuppressive to immunostimulatory. This more hospitable context allows PD-L1 blockade to improve the antitumor efficacy of T cells. The STING pathway is an attractive target in immuno-oncology as it can lead to potent adaptive antitumor response [36]. Recent work in a murine model of *Brca*-deficient ovarian cancer has demonstrated that the STING pathway is required for the dendritic cell-mediated antitumor activity of PARP inhibitors [37]. Interestingly, in that study STING activation also synergized with PD-1 checkpoint blockade therapy. In our study, chemotherapy and immunotherapy contribute equally to STING activation, as evidenced by increased expression of IRF3 [38]. DNA-damaging chemotherapy can yield DNA fragments that translocate to the cytosol, where they activate cGAS, leading to production of 2′3’-cGAMP intracellularly [39]. Still, chemotherapy is insufficient to generate meaningful survival benefit, for which combination with immunotherapy is required.

While chemotherapy can lead to initial activation and maturation of dendritic cells as well as release of tumor antigens for a subsequent vaccinal effect following apoptosis of cancer cells [40], immunotherapy drives a significant increase in the number of dendritic cells and maintains their activation. We hypothesize that presentation of tumor-specific antigens by mature MHCII^+^ dendritic cells mediates the activation and stimulation of Th17 CD4^+^ T cells, which become the main driver of tumor growth delay [41]. Th17 cells display a great degree of plasticity, rendering them capable of acquiring functional characteristics of Th1 cells [42, 43]. Interestingly, previous studies examining the role of Th17 cells in antitumor immunity revealed that Th17-polarized cells were even more effective than Th1 cells in mediating rejection of large B16 melanomas [44].

CD4^+^ T cells acquire a highly antitumor phenotype upon combination therapy, as evidenced by an increased proportion of cells expressing activation markers (ICOS, PD-1, CD44). CD4^+^ T cells can eliminate cancer cells directly in a perforin/granzyme B-dependent manner or indirectly via myeloid cells and/or NK cells. While NK cells seem to show increased activation after combination treatment (PD-1, CD44) [45, 46], their numbers do not increase (Additional file 12: Figure S12). Notably, 13 days after initiation of combination therapy, far more CD4^+^ T cells express granzyme B and EOMES. Together with the upregulation of MHCII^+^ expression on cancer cells, this phenomenon hints at a direct cytotoxic activity for the CD4^+^ T cells. Such functionality has been previously proposed in a model of melanoma, wherein antitumor activity was solely dependent on transferred CD4^+^ T cells [28, 41].

The data also demonstrate a significant increase in FoxP3^+^ Treg cells with combination therapy. It has been shown that there is considerable plasticity among Th17 and Tregs, with both subsets able to transdifferentiate into the other [47, 48]. Indeed, we found that there is a small subset of CD4^+^ T cells that co-expresses both RORγt and FoxP3, which could represent a transient population [49] (Additional file 13: Figure S13). Likely, anti-tumor Th17 cells convert into Tregs as the tumor progresses and prevent long-term survival in mice treated with the triple combination therapy. However, future studies will have to address this issue in more detail and investigate the Th17-Treg cell plasticity in this model. Potentially, addition of an CTLA-4 antibody that targets these cells could further improve survival. While past research has often focused predominantly on the immunosuppressive properties of CD4^+^ T cells [50], the data presented herein underline the complexity of CD4^+^ T cell plasticity and support the importance of conducting further research on exploiting the antitumor function of CD4^+^ T cells in immuno-oncology.

Given the growing numbers of clinicals trials involving combination therapy, our work on the temporal interplay of chemotherapy and immunotherapy is highly relevant. It has been previously reported that paclitaxel and carboplatin chemotherapy augments anti-tumor immunity through a powerful cytotoxic T lymphocyte response and proposed a period of 12–14 days after chemotherapy as the optimal opportunity for T cell-focused immunotherapy [51]. However, that work is mainly based on analysis of in vitro cultured T cells isolated from human ovarian cancer patients and this context fails to recapitulate the complex interactions in the tumor microenvironment and the immunosuppressive influence of myeloid cells. Furthermore, the selection of their measurement timepoints misses the early effects of chemotherapy. Our work shows the acute effects of chemotherapy on the innate immune system, and that the benefits of combination therapy are lost when administration of immunotherapy is delayed. It therefore stands to reason that immunotherapies targeting the innate immune system should be administered concomitant with chemotherapy. Still, consistent with the work of Wu et al., our results and unpublished data also show that T cells were not stimulated by chemotherapy during the first 7 days after chemotherapy, hinting that – unlike anti-IL-10 and 2′3’-cGAMP – anti-PD-L1 dosing could be delayed until the T cell compartment is fully primed without compromising survival benefits.

A more sequential, serial delivery of immunotherapy could possibly also reduce the likelihood and severity of adverse events, which have been frequently reported with administration of combination immunotherapy in the clinic [52]. Although we did not detect any toxicity among mice following administration of five different drugs in our study, this will likely be a greater concern among patients.

Still, a Phase III clinical trial in newly diagnosed advanced ovarian cancer is currently administering five different drugs, including carboplatin, paclitaxel, and immunotherapy [53].

The fact that the combination of chemotherapy plus anti-IL-10, 2′3’-cGAMP, and anti-PD-L1 was effective not only against ovarian cancer but also against lung cancer, which presents a completely different tumor microenvironment, suggests that this combination approach could potentially be employed in a variety of tumors that have not responded to adaptive immunotherapy alone to date.

## Conclusions

In conclusion, we found a combination treatment of chemotherapy and immunotherapy that markedly prolongs survival in murine models of ovarian and lung cancer. The use of anti-IL-10, 2′3’-cGAMP, and anti-PD-L1 engages both the innate and adaptive arms of the immune system. Thereby, immunotherapy counteracts the immunosuppressive shift mediated by the myeloid cell population while chemotherapy effectively activated dendritic cells. Together, they increase the expression of pro-inflammatory molecules as well as the numbers of activated T cells and mature dendritic cells. The data indicate that survival benefit is strongly dependent on a mechanistically informed dosing schedule. On a cellular level, Th17 CD4^+^ T cells appear to be particularly important, and their effects are thought to be mediated directly via GZMB. We believe that these data support the utility of clinical trials for ovarian cancer patients that combine immunotherapies that target both innate and adaptive immunity. As importantly, they underscore the importance of tumor-reactive CD4^+^ T cells in mediating anti-tumor immunity. Finally, the complete loss of efficacy upon delayed or abbreviated administration of the immunotherapies highlights the need to be thoughtful about dosing regimens in the clinic.

## Additional files

Additional file 1: **Figure S1.** Chemotherapy prolongs survival but does not synergize with PD-1 checkpoint blockade. (**a**) Mice were inoculated with ID8-Vegf-Defb29 cancer cells. Eight days later, mice were injected with either vehicle or paclitaxel and carboplatin (Chemo). A Kaplan-Meier curve is shown. (**b**) Mice were inoculated with ID8-Vegf-Defb29 cancer cells. Eight days later, treatment with chemotherapy alone or chemotherapy and anti-PD-L1 checkpoint blockade was initiated. The number of mice per group (n) and median survival (ms) are listed. Experiments were performed with biological replicates once or twice. Statistics were calculated using the Log-rank (Mantel-Cox) test. **** *p* ≤ 0.0001. (PDF 94 kb)

Additional file 2: **Figure S2.** Functional enrichment analysis of unsorted cells highlights the importance of the innate immune system. Mice were inoculated with ID8-Vegf-Defb29 cancer cells. Eight days later, mice were injected with either vehicle or paclitaxel and carboplatin (Chemo). Two days thereafter, peritoneal cells were recovered and assessed by gene expression analysis. (**a**) The top 84 significantly upregulated genes after chemotherapy treatment with a fold change ≥1 were queried for their pathway interactions using the ToppGene Suite gene list enrichment analysis tool. (**b**) The genes induced by chemotherapy had a significant correlation with myeloid cell populations. (PDF 1020 kb)

Additional file 3: **Figure S3.** Gating strategy used in flow cytometric analysis of immune cells harvested from the peritoneal cavity after treatment. Flow cytometric data were analyzed using FlowJo software. (PDF 423 kb)

Additional file 4: **Figure S4.** Chemotherapy induces an increase in granulocytic but not monocytic MDSCs. Mice were inoculated orthotopically with ID8-Vegf-Defb29 ovarian cancer cells. Eight days later, the mice were injected with vehicle (Veh) or chemotherapy (Chemo). Two days later, peritoneal cells were harvested and assessed by flow cytometry. (**a**) Increased numbers of granulocytic MDSCs (Ly6G^+^Ly6C^+^) are observed following chemotherapy. (**b**) Numbers of monocytic MDSCs (Ly6G^−^Ly6C^+^) and their expression of immunosuppressive ARG1 and IL-10 are quantified. The experiment was performed twice with *n* = 4 biological replicates. Statistics were calculated using a two-sided unpaired t-test. Data are presented as mean ± SEM **** *p* ≤ 0.0001. (PDF 78 kb)

Additional file 5: **Figure S5.** Galectin3 is upregulated after chemotherapy treatment. Mice were treated with vehicle (Veh) or chemotherapy (Chemo) 8 days after ID8-Vegf-Defb29 tumor inoculation and peritoneal cells were assessed by flow cytometry 4 days after initiation of treatment. Histograms of Gal3^+^ expression on CD11b^+^ myeloid cells are shown and the mean fluorescence intensity was quantified on the right. Experiment was performed twice with *n* = 5 biological replicates. Statistics were calculated using a two-sided unpaired t-test. Data are presented as mean ± SEM ** *p* ≤ 0.01 (PDF 97 kb)

Additional file 6: **Figure S6.** Proportion of CD4^+^ and CD8^+^ T cells is not affected shortly after treatment. Peritoneal cells harvested from mice treated with vehicle (Veh); chemotherapy (Chemo); anti-IL-10, 2′3’-cGAMP, and anti-PD-L1 immunotherapy (IT); or both Chem and IT (Combo) were assessed by flow cytometry 4 days after initiation of treatment. Flow cytometry gating of subsets of CD4^+^ and CD8^+^ expressing CD3^+^ T cells are shown as scatter plots and quantified at right. Experiment was performed twice with *n* = 4 biological replicates. Statistics were calculated using one-way ANOVA with Tukey’s multiple comparisons test. Data are presented as mean ± SEM. (PDF 76 kb)

Additional file 7: **Figure S7.** Combination therapy increases the proportion of differentiated CD8^+^ T cells. Peritoneal cells harvested from mice treated with vehicle (Veh); chemotherapy (Chemo); anti-IL-10, 2′3’-cGAMP, and anti-PD-L1 immunotherapy (IT); or both Chemo and IT (Combo) were assessed by flow cytometry 4 days after initiation of treatment. Flow cytometry gating of subsets of CD69 and CD107a expressing CD8^+^ T cells are shown as scatter plots and quantified at right. Experiment was performed twice with *n* = 4 biological replicates. Statistics were calculated using one-way ANOVA with Tukey’s multiple comparisons test. Data are presented as mean ± SEM * *p* ≤ 0.05. (PDF 83 kb)

Additional file 8: **Figure S8.** Expression of IFNγ or PD-1 on T cells is not affected shortly after treatment. Peritoneal cells harvested from mice treated with vehicle (Veh); chemotherapy (Chemo); anti-IL-10, 2′3’-cGAMP, and anti-PD-L1 immunotherapy (IT); or both Chemo and IT (Combo) were assessed by flow cytometry 4 days after initiation of treatment. Flow cytometry gating of subsets of PD-1^+^ and IFNγ^+^ g CD4^+^ and CD8^+^ T cells are shown as scatter plots and quantified at right. Experiment was performed twice with *n* = 4 biological replicates. Statistics were calculated using one-way ANOVA with Tukey’s multiple comparisons test. Data are presented as mean ± SEM. (PDF 84 kb)

Additional file 9: **Figure S9.** Flow cytometry analysis confirms that CD11b^+^ myeloid cells, CD8a^+^ T cells, and CD4^+^ T cells are depleted following administration of appropriate antibodies**.** (**a**) Plots are shown for leukocytes isolated from blood after initiation of treatment. (**b**) Quantification of depletion is representative of *n* = 6 mice, and the experiment was performed twice. Statistics were calculated using a two-sided unpaired t-test. Data are presented as mean ± SEM **** *p* ≤ 0.0001. (PDF 114 kb)

Additional file 10: **Figure S10.** Tbet transcription factor is upregulated after immunotherapy. Peritoneal cells harvested from mice treated with vehicle (Veh); chemotherapy (Chemo); anti-IL-10, 2′3’-cGAMP, and anti-PD-L1 immunotherapy (IT); or both Chemo and IT (Combo) were assessed by flow cytometry 13 days after initiation of treatment. Bar graph shows quantification of flow cytometry gating of Tbet expression on CD4^+^ T cells. Experiment was performed twice with *n* = 4 biological replicates. Statistics were calculated using one-way ANOVA with Tukey’s multiple comparisons test. Data are presented as mean ± SEM *** *p* ≤ 0.001. (PDF 47 kb)

Additional file 11: **Figure S11.** Combination therapy increases the proportion of mature dendritic cells. Peritoneal cells harvested from mice treated with vehicle (Veh); chemotherapy (Chemo); anti-IL-10, 2′3’-cGAMP, and anti-PD-L1 immunotherapy (IT); or both Chemo and IT (Combo) were assessed by flow cytometry 13 days after initiation of treatment. Bar graph shows quantification of flow cytometry gating of MHCII/CD11c expression on CD45^+^ T cells. Experiment was performed twice with *n* = 4 biological replicates. Statistics were calculated using one-way ANOVA with Tukey’s multiple comparisons test. Data are presented as mean ± SEM *** *p* ≤ 0.001. (PDF 130 kb)

Additional file 12: **Figure S12.** Numbers of NK cells are not affected by combination therapy**.** Peritoneal cells harvested from mice treated with vehicle (Veh); chemotherapy (Chemo); anti-IL-10, 2′3’-cGAMP, and anti-PD-L1 immunotherapy (IT); or both Chemo and IT (Combo) were assessed by flow cytometry 13 days after initiation of treatment. Bar graph shows quantification of NK cells (NK1.1^+^CD3^−^) and their expression of activation makers CD44 and PD-1. Experiment was performed twice with *n* = 4 biological replicates. Statistics were calculated using one-way ANOVA with Tukey’s multiple comparisons test. Data are presented as mean ± SEM * *p* ≤ 0.05, ** *p* ≤ 0.01. (PDF 73 kb)

Additional file 13: **Figure S13.** Combination therapy increases a transient Th17/Treg cell population**.** Peritoneal cells harvested from mice treated with vehicle (Veh); chemotherapy (Chemo); anti-IL-10, 2′3’-cGAMP, and anti-PD-L1 immunotherapy (IT); or both Chemo and IT (Combo) were assessed by flow cytometry 13 days after initiation of treatment. Bar graph shows quantification of flow cytometry gating of RORγt/FoxP3 expression on CD4^+^ T cells. Experiment was performed twice with *n* = 4 biological replicates. Statistics were calculated using one-way ANOVA with Tukey’s multiple comparisons test. Data are presented as mean ± SEM * *p* ≤ 0.05, **** *p* ≤ 0.0001. (PDF 94 kb)

Additional file 14: **Table S1.** Antibodies used for flow cytometry experiments. (PDF 69 kb)

Additional file 15: **Table S2.** Summary of all triple immunotherapy combinations evaluated. (PDF 43 kb)

## Data Availability

The datasets used and/or analyzed during the current study are available from the corresponding author on reasonable request.

## Abbreviations

ARG1: Arginine
cGAMP: Cyclic guanosine monophosphate–adenosine monophosphate
cGAS: Cyclic GMP-AMP Synthase
Chemo: Chemotherapy
Combo: Combination therapy
CTLA4: Cytotoxic T lymphocyte–associated protein 4
DEFB29: Beta-defensin 29
FBS: Fetal bovine serum
FoxP3: Forkhead box P3
Gal3: Galectin 3
GZMB: Granzyme B
IACUC: Institutional Animal Care and Use Committee
ICOS: Inducible T-cell costimulatory
IFNs: Interferons
IT: Immunotherapy
LLC: Lewis Lung Carcinoma
MDSCs: Myeloid derived suppressor cells
MHCII: Major histocompatibility complex class II molecule
mm^3^: Cubic millimeter
ms: Medium survival
NK: Natural killer
PD-1: Programmed death 1
PD-L1: Programmed death ligand 1
RORγt: RAR-related orphan receptor gamma 2
STING: Stimulator of interferon genes
T-bet: T-box transcription factor
Th: T helper
Treg: Regulatory T cell
VEGF-A: Vascular endothelial growth factor A
Veh: Vehicle
