# Supplementary figures and images for: Combination therapy targeting both innate and adaptive immunity improves survival in a pre-clinical model of ovarian cancer
Source: J Immunother Cancer. 2019 Jul 30;7:199. doi: 10.1186/s40425-019-0654-5 (PMC6668091; doi:10.1186/s40425-019-0654-5)

Supplementary Figure 1.

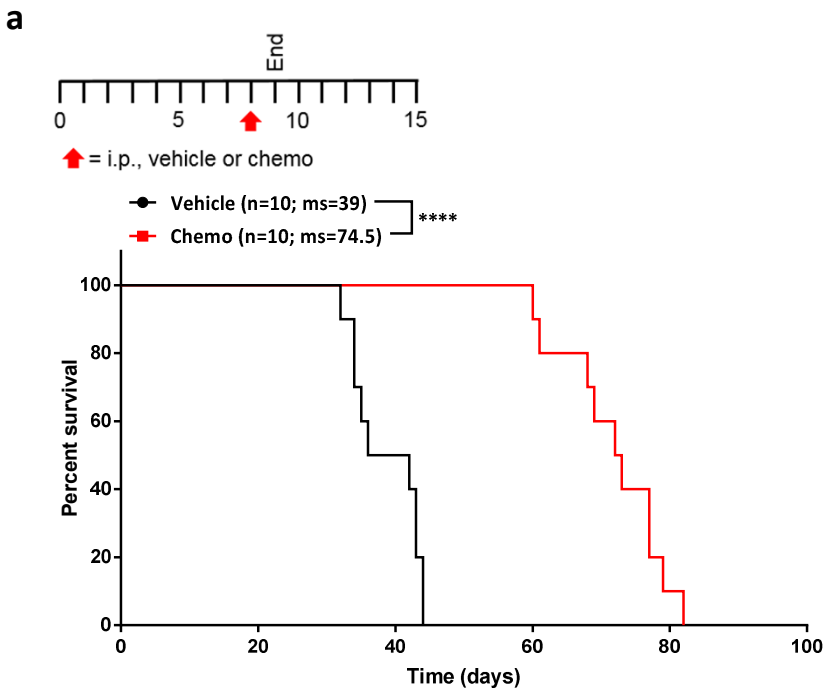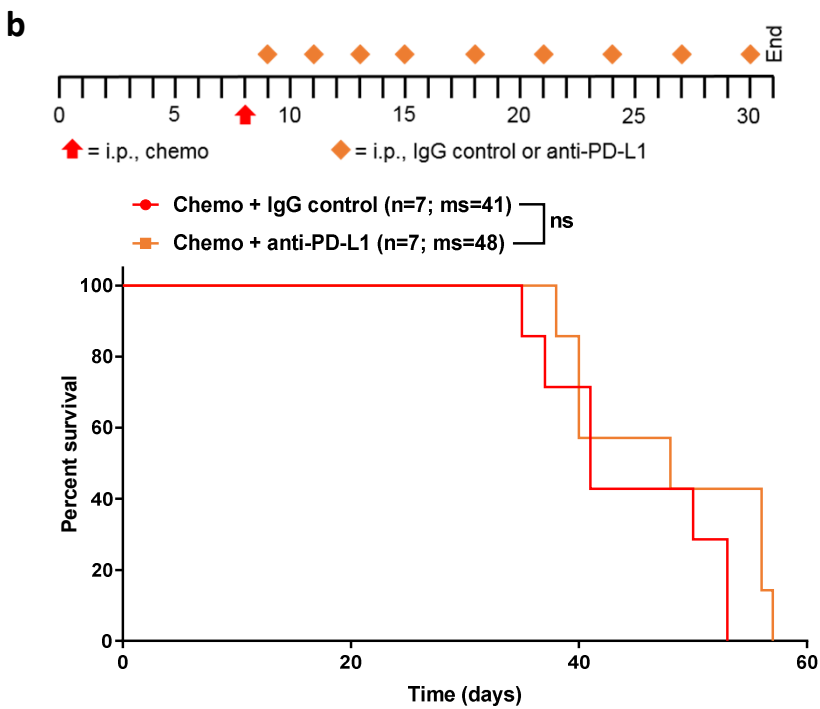

Supplement: Supplementary file 1 — Figure S1. Chemotherapy prolongs survival but does not synergize with PD-1 checkpoint blockade. (a) Mice were inoculated with ID8-Vegf-Defb29 cancer cells. Eight days later, mice were injected with either vehicle or paclitaxel and carboplatin (Chemo). A Kaplan-Meier curve is shown. (b) Mice were inoculated with ID8-Vegf-Defb29 cancer cells. Eight days later, treatment with chemotherapy alone or chemotherapy and anti-PD-L1 checkpoint blockade was initiated. The number of mice per group (n) and median survival (ms) are listed. Experiments were performed with biological replicates once or twice. Statistics were calculated using the Log-rank (Mantel-Cox) test. **** p ≤ 0.0001. (PDF 94 kb) [file 40425_2019_654_MOESM1_ESM.pdf]

Supplementary Figure 3.

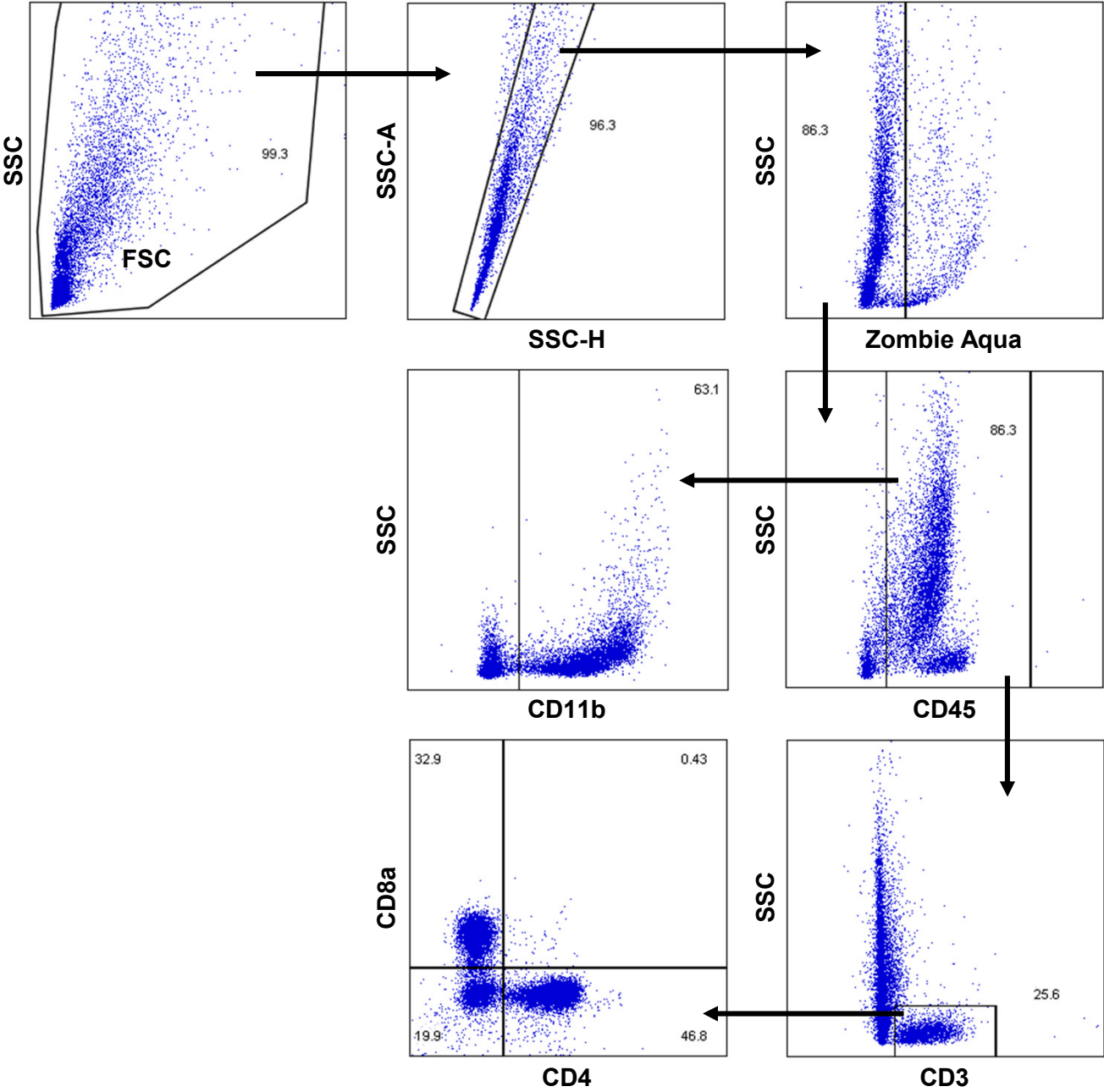

Supplement: Supplementary file 3 — Figure S3. Gating strategy used in flow cytometric analysis of immune cells harvested from the peritoneal cavity after treatment. Flow cytometric data were analyzed using FlowJo software. (PDF 423 kb) [file 40425_2019_654_MOESM3_ESM.pdf]

Supplementary Figure 4.

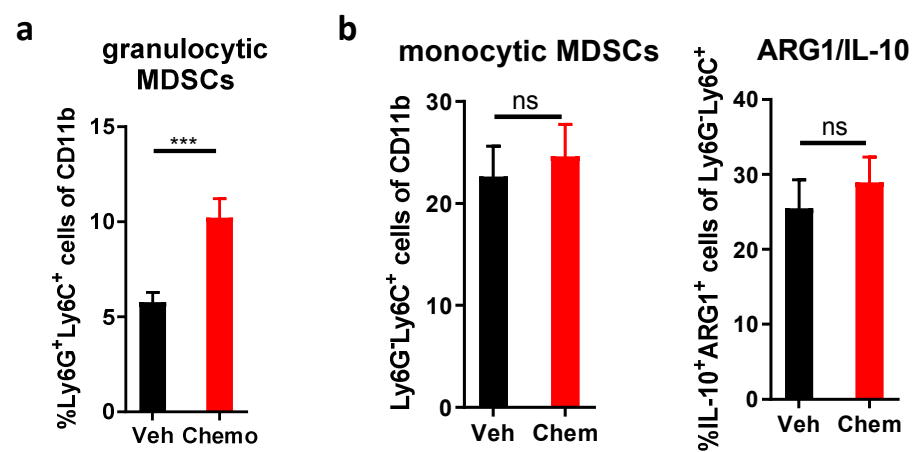

Supplement: Supplementary file 4 — Figure S4. Chemotherapy induces an increase in granulocytic but not monocytic MDSCs. Mice were inoculated orthotopically with ID8-Vegf-Defb29 ovarian cancer cells. Eight days later, the mice were injected with vehicle (Veh) or chemotherapy (Chemo). Two days later, peritoneal cells were harvested and assessed by flow cytometry. (a) Increased numbers of granulocytic MDSCs (Ly6G+Ly6C+) are observed following chemotherapy. (b) Numbers of monocytic MDSCs (Ly6G−Ly6C+) and their expression of immunosuppressive ARG1 and IL-10 are quantified. The experiment was performed twice with n = 4 biological replicates. Statistics were calculated using a two-sided unpaired t-test. Data are presented as mean ± SEM **** p ≤ 0.0001. (PDF 78 kb) [file 40425_2019_654_MOESM4_ESM.pdf]

Supplementary Figure 5.

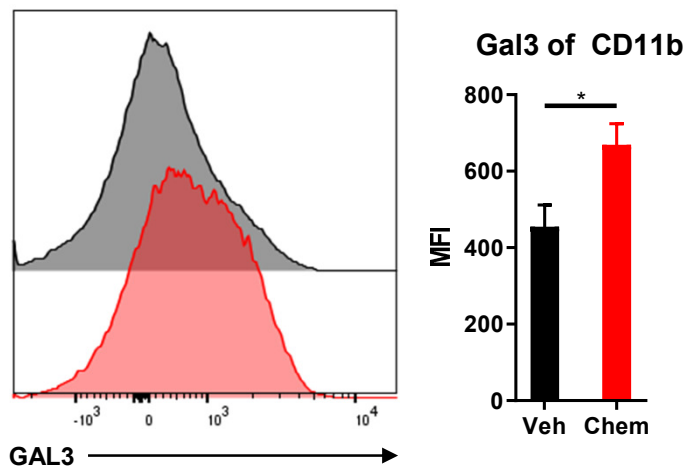

Supplement: Supplementary file 5 — Figure S5. Galectin3 is upregulated after chemotherapy treatment. Mice were treated with vehicle (Veh) or chemotherapy (Chemo) 8 days after ID8-Vegf-Defb29 tumor inoculation and peritoneal cells were assessed by flow cytometry 4 days after initiation of treatment. Histograms of Gal3+ expression on CD11b+ myeloid cells are shown and the mean fluorescence intensity was quantified on the right. Experiment was performed twice with n = 5 biological replicates. Statistics were calculated using a two-sided unpaired t-test. Data are presented as mean ± SEM ** p ≤ 0.01 (PDF 97 kb) [file 40425_2019_654_MOESM5_ESM.pdf]

Supplementary Figure 6.

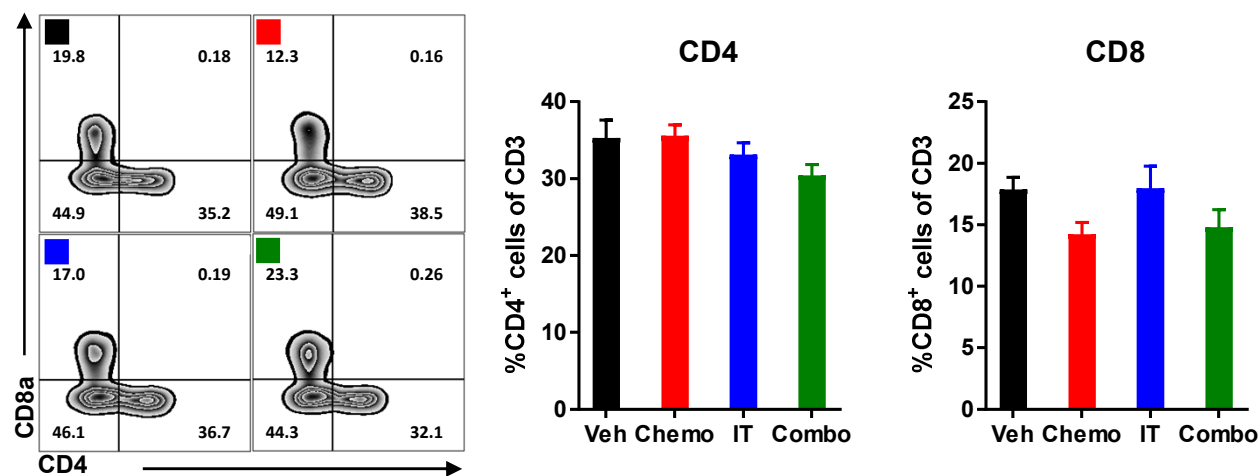

Supplement: Supplementary file 6 — Figure S6. Proportion of CD4+ and CD8+ T cells is not affected shortly after treatment. Peritoneal cells harvested from mice treated with vehicle (Veh); chemotherapy (Chemo); anti-IL-10, 2′3’-cGAMP, and anti-PD-L1 immunotherapy (IT); or both Chem and IT (Combo) were assessed by flow cytometry 4 days after initiation of treatment. Flow cytometry gating of subsets of CD4+ and CD8+ expressing CD3+ T cells are shown as scatter plots and quantified at right. Experiment was performed twice with n = 4 biological replicates. Statistics were calculated using one-way ANOVA with Tukey’s multiple comparisons test. Data are presented as mean ± SEM. (PDF 76 kb) [file 40425_2019_654_MOESM6_ESM.pdf]

Supplementary Figure 7.

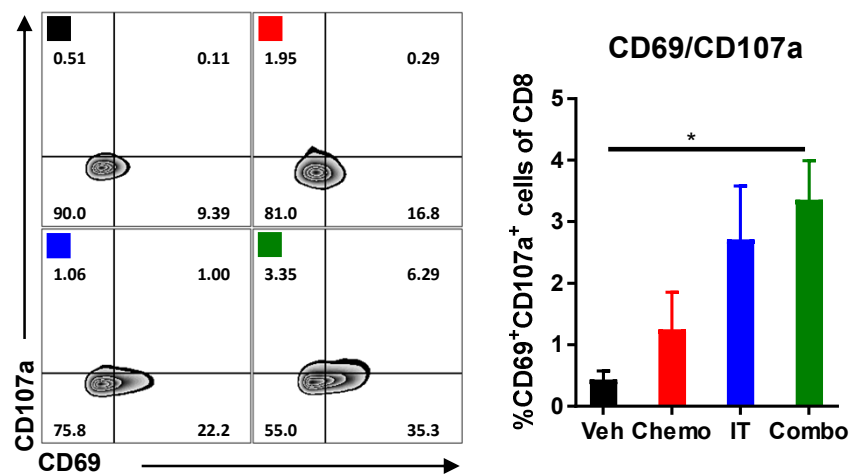

Supplement: Supplementary file 7 — Figure S7. Combination therapy increases the proportion of differentiated CD8+ T cells. Peritoneal cells harvested from mice treated with vehicle (Veh); chemotherapy (Chemo); anti-IL-10, 2′3’-cGAMP, and anti-PD-L1 immunotherapy (IT); or both Chemo and IT (Combo) were assessed by flow cytometry 4 days after initiation of treatment. Flow cytometry gating of subsets of CD69 and CD107a expressing CD8+ T cells are shown as scatter plots and quantified at right. Experiment was performed twice with n = 4 biological replicates. Statistics were calculated using one-way ANOVA with Tukey’s multiple comparisons test. Data are presented as mean ± SEM * p ≤ 0.05. (PDF 83 kb) [file 40425_2019_654_MOESM7_ESM.pdf]

Supplementary Figure 8.

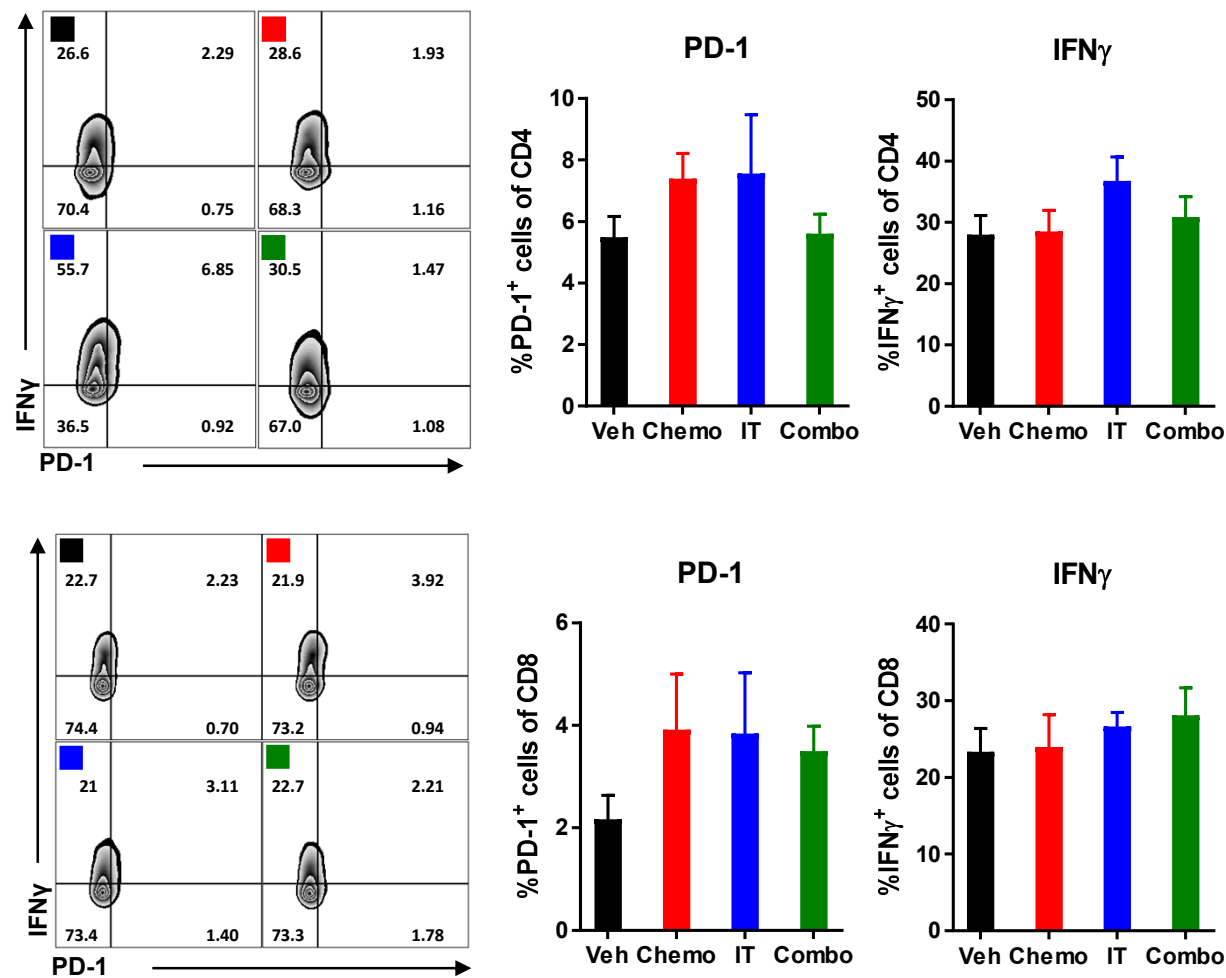

Supplement: Supplementary file 8 — Figure S8. Expression of IFNγ or PD-1 on T cells is not affected shortly after treatment. Peritoneal cells harvested from mice treated with vehicle (Veh); chemotherapy (Chemo); anti-IL-10, 2′3’-cGAMP, and anti-PD-L1 immunotherapy (IT); or both Chemo and IT (Combo) were assessed by flow cytometry 4 days after initiation of treatment. Flow cytometry gating of subsets of PD-1+ and IFNγ+ g CD4+ and CD8+ T cells are shown as scatter plots and quantified at right. Experiment was performed twice with n = 4 biological replicates. Statistics were calculated using one-way ANOVA with Tukey’s multiple comparisons test. Data are presented as mean ± SEM. (PDF 84 kb) [file 40425_2019_654_MOESM8_ESM.pdf]

Supplementary Figure 9.

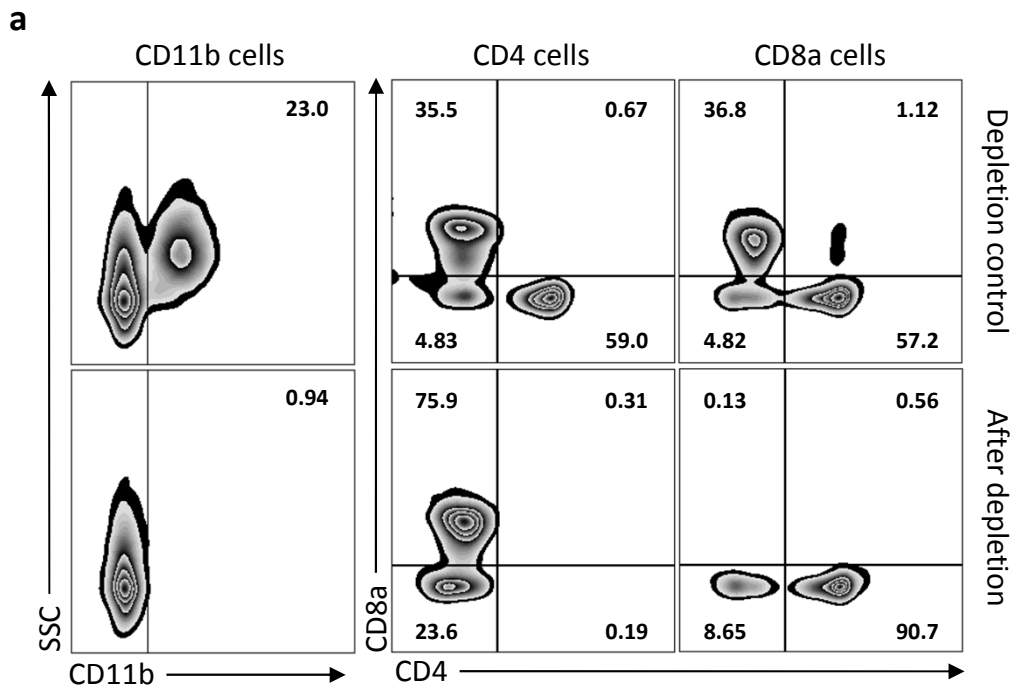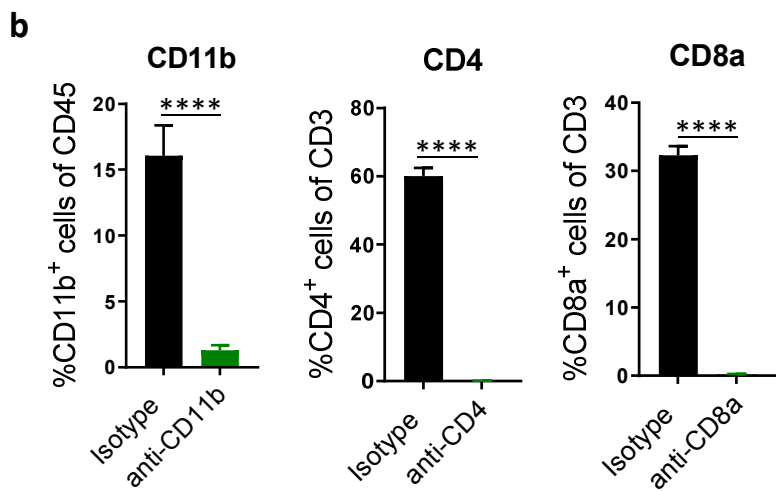

Supplement: Supplementary file 9 — Figure S9. Flow cytometry analysis confirms that CD11b+ myeloid cells, CD8a+ T cells, and CD4+ T cells are depleted following administration of appropriate antibodies. (a) Plots are shown for leukocytes isolated from blood after initiation of treatment. (b) Quantification of depletion is representative of n = 6 mice, and the experiment was performed twice. Statistics were calculated using a two-sided unpaired t-test. Data are presented as mean ± SEM **** p ≤ 0.0001. (PDF 114 kb) [file 40425_2019_654_MOESM9_ESM.pdf]

Supplementary Figure 10.

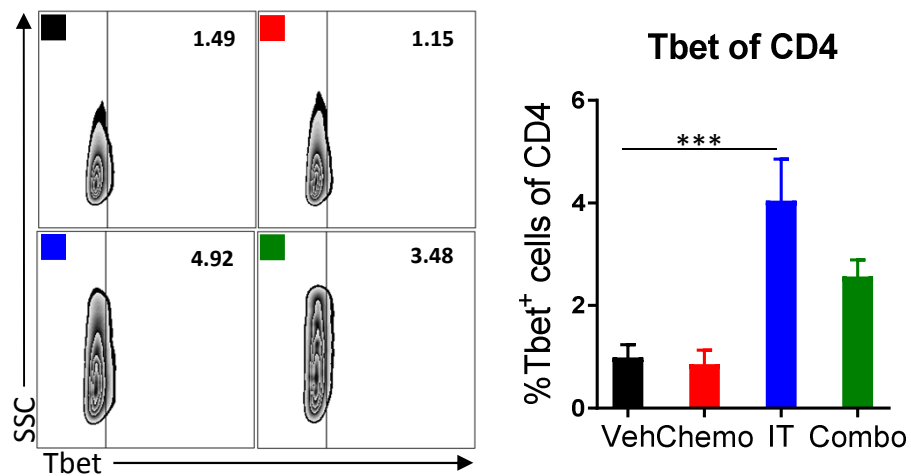

Supplement: Supplementary file 10 — Figure S10. Tbet transcription factor is upregulated after immunotherapy. Peritoneal cells harvested from mice treated with vehicle (Veh); chemotherapy (Chemo); anti-IL-10, 2′3’-cGAMP, and anti-PD-L1 immunotherapy (IT); or both Chemo and IT (Combo) were assessed by flow cytometry 13 days after initiation of treatment. Bar graph shows quantification of flow cytometry gating of Tbet expression on CD4+ T cells. Experiment was performed twice with n = 4 biological replicates. Statistics were calculated using one-way ANOVA with Tukey’s multiple comparisons test. Data are presented as mean ± SEM *** p ≤ 0.001. (PDF 47 kb) [file 40425_2019_654_MOESM10_ESM.pdf]

Supplementary Figure 11.

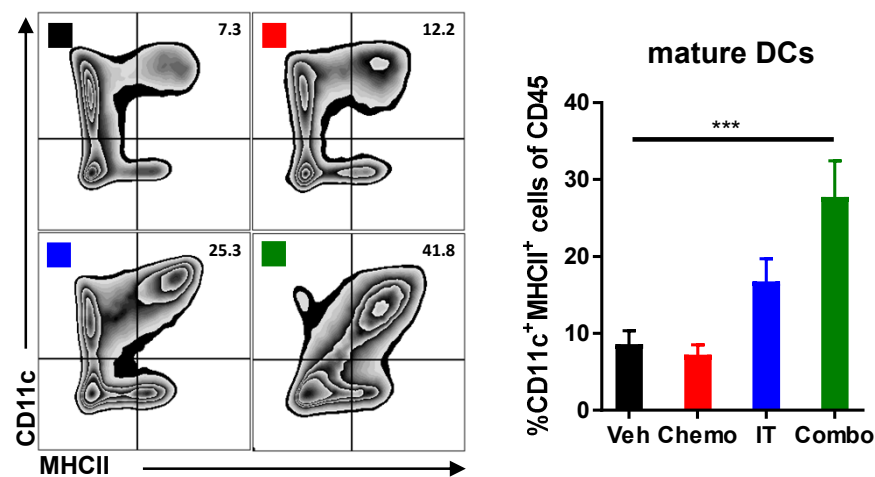

Supplement: Supplementary file 11 — Figure S11. Combination therapy increases the proportion of mature dendritic cells. Peritoneal cells harvested from mice treated with vehicle (Veh); chemotherapy (Chemo); anti-IL-10, 2′3’-cGAMP, and anti-PD-L1 immunotherapy (IT); or both Chemo and IT (Combo) were assessed by flow cytometry 13 days after initiation of treatment. Bar graph shows quantification of flow cytometry gating of MHCII/CD11c expression on CD45+ T cells. Experiment was performed twice with n = 4 biological replicates. Statistics were calculated using one-way ANOVA with Tukey’s multiple comparisons test. Data are presented as mean ± SEM *** p ≤ 0.001. (PDF 130 kb) [file 40425_2019_654_MOESM11_ESM.pdf]

Supplementary Figure 12.

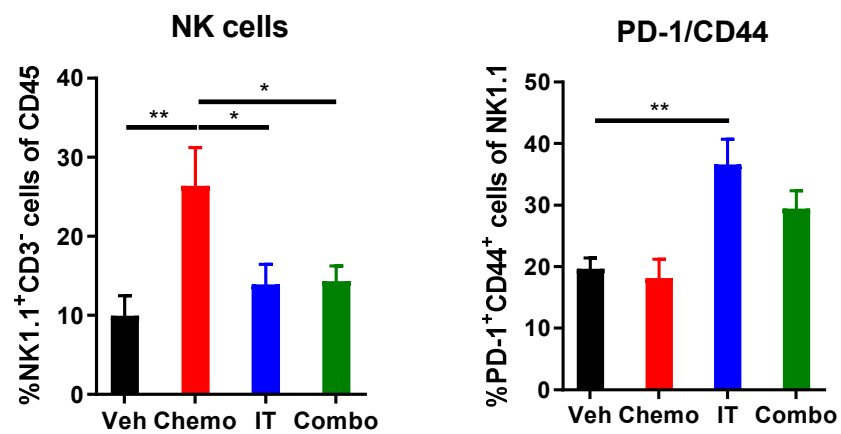

Supplement: Supplementary file 12 — Figure S12. Numbers of NK cells are not affected by combination therapy. Peritoneal cells harvested from mice treated with vehicle (Veh); chemotherapy (Chemo); anti-IL-10, 2′3’-cGAMP, and anti-PD-L1 immunotherapy (IT); or both Chemo and IT (Combo) were assessed by flow cytometry 13 days after initiation of treatment. Bar graph shows quantification of NK cells (NK1.1+CD3−) and their expression of activation makers CD44 and PD-1. Experiment was performed twice with n = 4 biological replicates. Statistics were calculated using one-way ANOVA with Tukey’s multiple comparisons test. Data are presented as mean ± SEM * p ≤ 0.05, ** p ≤ 0.01. (PDF 73 kb) [file 40425_2019_654_MOESM12_ESM.pdf]

Supplementary Figure 13.

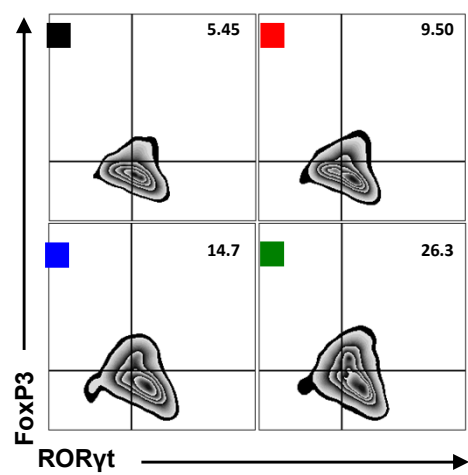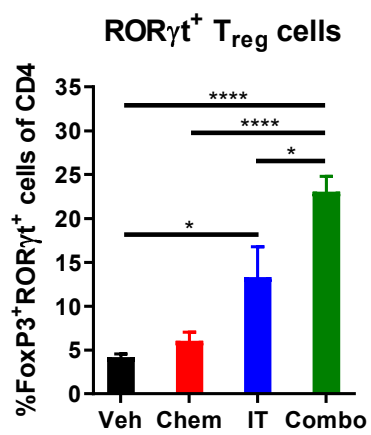

Supplement: Supplementary file 13 — Figure S13. Combination therapy increases a transient Th17/Treg cell population. Peritoneal cells harvested from mice treated with vehicle (Veh); chemotherapy (Chemo); anti-IL-10, 2′3’-cGAMP, and anti-PD-L1 immunotherapy (IT); or both Chemo and IT (Combo) were assessed by flow cytometry 13 days after initiation of treatment. Bar graph shows quantification of flow cytometry gating of RORγt/FoxP3 expression on CD4+ T cells. Experiment was performed twice with n = 4 biological replicates. Statistics were calculated using one-way ANOVA with Tukey’s multiple comparisons test. Data are presented as mean ± SEM * p ≤ 0.05, **** p ≤ 0.0001. (PDF 94 kb) [file 40425_2019_654_MOESM13_ESM.pdf]
