# Supplementary material for: Combination therapy targeting both innate and adaptive immunity improves survival in a pre-clinical model of ovarian cancer
Source: J Immunother Cancer. 2019 Jul 30;7:199. doi: 10.1186/s40425-019-0654-5 (PMC6668091; doi:10.1186/s40425-019-0654-5)
Supplement: Supplementary file 2 — Figure S2. Functional enrichment analysis of unsorted cells highlights the importance of the innate immune system. Mice were inoculated with ID8-Vegf-Defb29 cancer cells. Eight days later, mice were injected with either vehicle or paclitaxel and carboplatin (Chemo). Two days thereafter, peritoneal cells were recovered and assessed by gene expression analysis. (a) The top 84 significantly upregulated genes after chemotherapy treatment with a fold change ≥1 were queried for their pathway interactions using the ToppGene Suite gene list enrichment analysis tool. (b) The genes induced by chemotherapy had a significant correlation with myeloid cell populations. (PDF 1020 kb) [file 40425_2019_654_MOESM2_ESM.pdf]

Supplementary Figure 2.

a

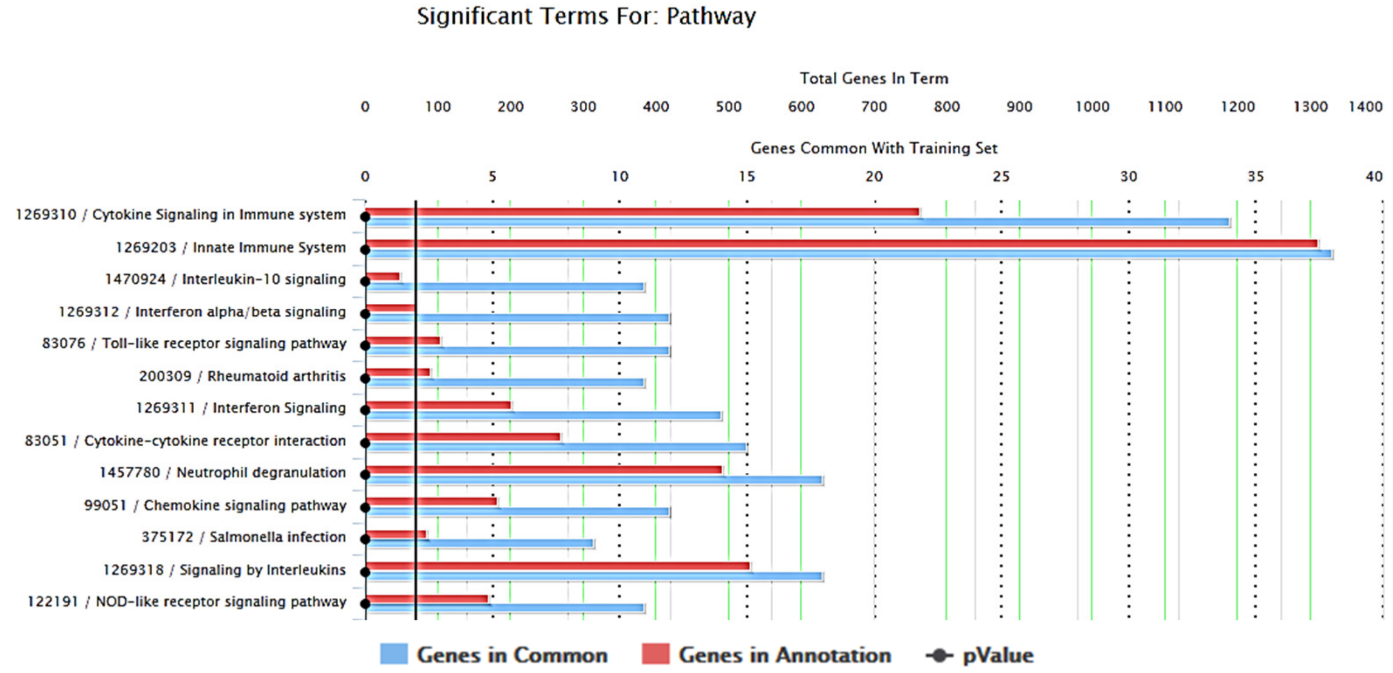

b

	ID	Name	pValue	FDR B&H	Genes from Input	Genes in Annotation
1	M2568	Genes up-regulated in lung tissue upon LPS aspiration with mechanical ventilation (MV) compared to control (PBS aspiration without MV).	8.832E-38	6.185E-34	25	128
2	19795415-Table1	Mouse Lung Beisiegel09 262genes	9.697E-29	3.395E-25	23	207
3	16479545-Table2	Mouse Lung not cancer Kahnert06 70genes	3.788E-28	8.842E-25	16	50
4	12446012-Table2	Mouse Immune Carmody02 151genes	1.415E-25	2.478E-22	18	112
5	20220088-SuppTable2a	Human Immune Allen10 106genes	2.142E-24	3.000E-21	17	103
6	M3302	Genes down-regulated in comparison of unstimulated peripheral blood mononuclear cells (PBMC) 3 days after stimulation with YF17D vaccine versus PBMC 7 days after the stimulation.	4.178E-24	4.877E-21	20	199
7	20460173-ImmPortAntimicrobials	Human Immune Kong10 544genes ImmPort Antimicrobials	3.231E-23	3.233E-20	25	469
8	M3010	Genes transcriptionally modulated in the blood of multiple sclerosis patients in response to subcutaneous treatment with recombinant IFNB1 [GeneID = 3456].	3.775E-23	3.304E-20	16	95
9	M14829	Genes down-regulated in hematopoietic precursor cells conditionally expressing HOXA9 and MEIS1 [GeneID=3205;4211].	8.957E-23	6.970E-20	15	77
10	M3296	Genes down-regulated in comparison of unstimulated peripheral blood mononuclear cells (PBMC) 1 day after stimulation with YF17D vaccine versus PBMC 7 days after the stimulation.	1.744E-22	1.036E-19	19	199
11	M5932	Genes defining inflammatory response.	1.922E-22	1.036E-19	19	200
12	M5913	Genes up-regulated in response to IFNG [GeneID=3458].	1.922E-22	1.036E-19	19	200
13	M7237	Genes down-regulated in thymus cortical regions: subcapsular versus central cortical.	1.922E-22	1.036E-19	19	200
14	16081686-SuppTable2	Human Lymphoma Tome05 357genes	3.171E-21	1.586E-18	17	155
15	M3288	Genes down-regulated in comparison of unstimulated peripheral blood mononuclear cells (PBMC) versus PBMC 7 days after stimulation with YF17D vaccine.	7.418E-21	3.056E-18	18	200
16	M9572	Genes up-regulated in cells from peripheral lymph nodes: T reg versus T conv.	7.418E-21	3.056E-18	18	200
17	M7320	Genes up-regulated in HMC-1 (mast leukemia) cells: incubated with the peptide ALL1 and then treated with Cl-IB-MECA [PubChem=3035850] versus stimulation by T cell membranes.	7.418E-21	3.056E-18	18	200
18	M1430	Genes defining differentiation potential of the bipotential myeloid cell line FDB.	9.472E-21	3.685E-18	17	165
19	17174972-TableS1	Human Zola07 426genes CellDifferentiationMarkers	1.089E-20	4.014E-18	21	345
20	M1920	Genes forming the macrophage-enriched metabolic network (MEMN) claimed to have a causal relationship with the metabolic syndrom traits.	1.273E-20	4.458E-18	32	1210
