# Supplementary material for: Combination therapy targeting both innate and adaptive immunity improves survival in a pre-clinical model of ovarian cancer
Source: J Immunother Cancer. 2019 Jul 30;7:199. doi: 10.1186/s40425-019-0654-5 (PMC6668091; doi:10.1186/s40425-019-0654-5)
Supplement: Supplementary file 14 — Table S1. Antibodies used for flow cytometry experiments. (PDF 69 kb) [file 40425_2019_654_MOESM14_ESM.pdf]

Supplementary Table S1. Antibodies used for flow cytometry experiments

Antigen	Conjugate	Clone	Vendor
Arg1	APC	Met1-Lys322	R&D Systems
B220	PE	RA3-6B2	BioLegend
CD107a	APC/Cy7	1D4B	BioLegend
CD11b	PE/Cy5	M1/70	BioLegend
CD11c	BV421	N418	BioLegend
CD3	FITC	17A2	BioLegend
CD3	APC	17A2	BioLegend
CD4	PE/Cy5	GK1.5	BioLegend
CD44	PE/Cy7	IM7	BioLegend
CD45	AF 700	30-F11	BioLegend
CD69	BV421	H1.2F3	BioLegend
CD8a	FITC	5H10-1	BioLegend
CD8a	PE-Cy7	53-6.7	BioLegend
CD80	PE/Cy5	16-10A1	BioLegend
CD86	BV605	GL-1	BioLegend
EOMES	AF488	Met1-Ser126	R&D Systems
F4/80	PerCP/Cy5.5	BM8	BioLegend
FoxP3	Pacific Blue	MF-14	BioLegend
Gal3	PE	Gal397	BioLegend
GZMB	PE/Cy7	QA16A02	BioLegend
ICOS	PE	7E.17G9	BioLegend
IFNγ	PE-Dazzle 594	XMG1.2	BioLegend
IL-10	PE-Cy7	JES5-16E3	BioLegend
IL-2	Pacific Blue	JES6-5H4	BioLegend
IRF3	AF 647	D601M	CST
Ly6C	AF700	HK1.4	BioLegend
Ly6G	PerCP	1A8	BioLegend
MHCII	PacificBlue	M5/114.15.2	BioLegend
NKp46	PE	29A1.4	BioLegend
PD-1	BV605	29F.1A12	BioLegend
RORγt	PE	Met1-Arg10	R&D Systems
Tbet	BV605	4B10	BioLegend
