# Supplementary material for: Combination therapy targeting both innate and adaptive immunity improves survival in a pre-clinical model of ovarian cancer
Source: J Immunother Cancer. 2019 Jul 30;7:199. doi: 10.1186/s40425-019-0654-5 (PMC6668091; doi:10.1186/s40425-019-0654-5)
Supplement: Supplementary file 15 — Table S2. Summary of all triple immunotherapy combinations evaluated. (PDF 43 kb) [file 40425_2019_654_MOESM15_ESM.pdf]

Supplementary Table S2. Summary of all triple immunotherapy combinations evaluated

Combinations	Chemo	aIL-10	GR-MD-02	a4-1BB	2'3'-cGAMP	gemcitabine	aPD-L1	Significance	p-value
a								ns	0.4832
b								ns	0.2408
c								****	<0.0001
d								**	0.0049
e								****	<0.0001
